# Development of a prognostic model based on lysosome-related genes for ovarian cancer: insights into tumor microenvironment, mutation patterns, and personalized treatment strategies

**DOI:** 10.1186/s12935-024-03586-w

**Published:** 2024-12-19

**Authors:** Ran Sun, Siyi Li, Wanlu Ye, Yanming Lu

**Affiliations:** Department of Obstetrics and Gynecology, Shengjing Hospital of China Medical University, Shenyang, 110022 China

**Keywords:** Ovarian cancer, Lysosome, Prognosis, Tumor microenvironment, Risk signature, qRT-PCR

## Abstract

**Background:**

Ovarian cancer (OC) is often associated with an unfavorable prognosis. Given the crucial involvement of lysosomes in tumor advancement, lysosome-related genes (LRGs) hold promise as potential therapeutic targets.

**Methods:**

To identify differentially expressed lysosome-related genes (DE-LRGs), we performed a matching analysis between differentially expressed genes (DEGs) in OC and the pool of LRGs. Genes with prognostic significance were analyzed using multiple regression analyses to construct a prognostic risk signature. The model's efficacy was validated through survival analysis in various cohorts. We further explored the model's correlation with clinical attributes, tumor microenvironment (TME), mutational patterns, and drug sensitivity. The quantitative real-time polymerase chain reaction (qRT-PCR) validated gene expression in OC cells.

**Results:**

A 10-gene prognostic risk signature was established. Survival analysis confirmed its predictive accuracy across cohorts. The signature served as an independent prognostic element for OC. The high-risk and low-risk groups demonstrated notable disparities in terms of immune infiltration patterns, mutational characteristics, and sensitivity to therapeutic agents. The qRT-PCR results corroborated and validated the findings obtained from the bioinformatic analyses.

**Conclusions:**

We devised a 10-LRG prognostic model linked to TME, offering insights for tailored OC treatments.

**Supplementary Information:**

The online version contains supplementary material available at 10.1186/s12935-024-03586-w.

## Introduction

Ovarian cancer (OC) is a prevalent neoplasm within gynecology in the world. Current projections anticipate that the United States will witness 19,710 newly diagnosed incidences of OC with a potential of 13,270 fatalities in 2023 [1]. Recent therapeutic advancements involving a comprehensive approach combining surgical procedures as well as chemotherapeutic, targeted therapeutic, and immunotherapeutic interventions have yielded substantial survival enhancements for OC patients [2]. Due to the absence of early symptomatology associated with OC, the identification of an effective remedy becomes challenging. As a result, nearly 70% of diagnosed patients encounter the disease during its advanced stages, thereby posing obstacles to the implementation of radical therapy [3]. Additionally, the recurrence of the illness and the emergence of drug resistance are frequent occurrences, which ultimately contribute to an unfavorable overall prognosis. Consequently, the discovery of novel biomarkers is of utmost importance for early-stage prognostic prediction in OC.

Lysosomes, encompassing ~ 60 distinct acidic hydrolases, are vital to degrading cellular waste and maintaining homeostasis [4, 5]. They regulate digestion via endocytosis and autophagy, and play a role in metabolic signaling, particularly through the mammalian target of rapamycin complex 1 (mTORC1) and adenosine monophosphate-activated protein kinase (AMPK) at the lysosomal membrane [5–7]. Extensive research findings have elucidated the pivotal role of lysosomes in the etiology and progression of tumorigenesis, profoundly influencing cellular processes such as tumor cell autophagy, apoptosis, necrosis, and ferroptosis [8]. Moreover, oncogenic transformation induces alterations in both lysosomal volume and subcellular position [9]. In particular, lysosomes undergo translocation from the perinuclear region to the peripheral cytoplasm, leading to an enhanced level of lysosomal exocytosis. Consequently, the heightened secretion of acid hydrolases can contribute to the degradation of the extracellular matrix, fostering enhanced cellular motility, migration, and invasion [8, 10]. Notably, the pursuit of lysosome-targeting drugs has gained momentum within clinical investigation and holds great potential in cancer treatment [11, 12]. Regarding OC, substantial advancement has been achieved in the exploration of lysosome-related genes (LRGs). Lysosome-associated membrane protein 1 (LAMP1), located in lysosomal membranes, is significantly overexpressed in OC and is linked to reduced survival, making it a potential prognostic marker and therapeutic target [13]. GBA, the genetic determinant for β-glucosidase, manifests as a lysosomal hydrolase exhibiting upregulated expression levels within OC specimens. Intriguingly, augmented GBA expression does not exhibit a notable influence on tumoral growth and migratory capacities. However, it significantly compromises the therapeutic effectiveness of cisplatin in OC cell lines [14]. This suggests that GBA could be a target to counteract cisplatin resistance. Nevertheless, a comprehensive assessment of the precise involvement of LRGs in determining the prognosis of OC warrants further exploration.

The tumor microenvironment (TME) encompasses the intracellular milieu in which tumor cells originate and thrive, comprising a diverse array of cellular and non-cellular constituents that profoundly impact tumor progression [15]. Notably, the lysosomal membrane-localized vacuolar H + -ATPases (V-ATPases) have demonstrated their capability to modulate the TME through the efflux of protons (H+) into the extracellular domain[16]. Lysosomes have an intrinsic role in modulating the behavior of tumor-associated macrophages, fibroblasts, and T cells, thereby exerting regulatory control over tumor progression [17]. Consequently, our objective was to develop a novel prognostic signature for OC by utilizing LRGs. Furthermore, our investigation aimed to explore the correlation between this prognostic signature and the TME, providing novel insights into the diagnosis and therapeutic approaches for this disease.

Our research is focused on the development of a new prognostic signature for OC, utilizing publicly available databases and focusing on the inclusion of LRGs. Subsequently, we assessed the interrelationship between this prognostic signature, the tumor microenvironment, and the gene mutational landscape, while concurrently exploring potential therapeutic candidates capable of exerting favorable effects in OC. Through our comprehensive analyses, we not only deepen the comprehension surrounding the intricate association between LRGs and OC prognosis but also elucidate novel avenues for OC treatment.

## Materials and methods

### Data acquisition and collection

Gene expression profiles, clinicopathological information, and somatic mutation data of OC patients were retrieved and downloaded from the Cancer Genome Atlas database (TCGA; https://portal.gdc.cancer.gov). And we obtained the expression values of healthy ovarian tissues from Genotype-Tissue Expression database (GTEx; https://www.gtexportal.org/home/datasets) as a suitable surrogate. To conduct a comprehensive evaluation of LRGs, we obtained a curated set of 876 LRGs from the Gene Ontology consortium (GO; http://geneontology.org/).

### Functional enrichment and PPI network analysis of LRGs

Metascape (http://metascape.org) serves as a robust computational platform facilitating comprehensive gene functional annotation and analysis [18]. To unravel and comprehend the diverse biological functions exhibited by LRGs, we employed Metascape as an efficacious tool to perform functional enrichment analysis of LRGs. Next, we examined the protein–protein interactions (PPI) among LRGs using the STRING database [19] (https://cn.string-db.org/) with a stringent confidence threshold of 0.900.

### Differential expression analysis

The gene expression profiles derived from the TCGA and GTEx datasets were subjected to a log_2_(FPKM + 1) transformation to achieve normalization, thereby successfully mitigating batch effects. To identify differentially expressed genes between tumor and normal samples, we used the "limma" R package (version 3.60.4), applying criteria that include |log_2_ fold change (FC)|> 1 and false discovery rate (FDR) < 0.05. By employing the "Venn" R package (version 1.2.0), we identified the intersection between the differentially expressed genes (DEGs) and the LRGs, facilitating the identification of differentially expressed LRGs (DE-LRGs) specific to OC.

### Development and verification of a prognostic signature associated with lysosomal function

A random partitioning of the TCGA cohort resulted in the formation of two distinct cohorts, namely the TCGA training cohort (n = 186) and the TCGA testing cohort (n = 181). Subsequently, the DE-LRGs were subjected to univariate Cox regression analysis, aiming to detect genes with significant prognostic associations (p < 0.05). To mitigate the potential risks of overfitting, a least absolute shrinkage and selection operator (LASSO) Cox regression analysis was employed to refine the independent variables. Subsequently, a robust risk signature was constructed by selecting genes significantly associated with prognosis through multivariate Cox regression analysis. The risk score was calculated by incorporating the gene expression levels with the corresponding regression coefficients, as indicated by the following formula:

$$\text{Risk score}=\sum_{i=1}^{n}{coef}_{i}\times {expr}_{i}.$$

Within the provided equation, the symbol "coef" signifies the regression coefficient corresponding to each particular gene, while "expr" represents the expression level of an individual gene. Additionally, "n" denotes the total number of genes taken into consideration during the analysis.

By utilizing the median risk score as the threshold, the patient population from the training cohort, test cohort, and TCGA cohort was classified into high-risk and low-risk groups. The subsequent assessment of survival disparity between these groups was accomplished by comparing the Kaplan–Meier survival curves. To ascertain the prognostic effectiveness of the developed signature, receiver operating characteristic (ROC) curve analysis was conducted, and the corresponding area under the curve (AUC) was calculated. Furthermore, principal component analysis [20] was employed to visually illustrate the distribution patterns of the entire gene set, the LRG subset, as well as the subset of 10 LRGs comprising the prognostic signature.

### Independent prognostic assessment and stratification analysis

To evaluate the risk score's potential as an independent prognostic factor, we performed univariate and multivariate Cox regression analyses. The risk score, along with other clinical characteristics, was used to develop a nomogram via the "rms" R package (version 6.4-1) [21]. The nomogram's predictive performance was evaluated with calibration curves. Additionally, stratified analyses were performed to investigate the predictive capacity of the risk score in patients with age > 65 years, age ≤ 65 years, grade G2 + G3, stage III + IV, recurrence, and disease progression.

### Analysis of immune infiltration patterns

The Estimation of STromal and Immune cells in MAlignant Tumors using Expression data (ESTIMATE) algorithm [22] was applied to determine the ESTIMATE score, immune score, and stromal score for each sample. Additionally, the infiltration of immune cells in tumor tissues was estimated using a range of algorithms such as TIMER, CIBERSORT, CIBERSORT-ABS, QUANTISEQ, MCP-COUNTER, XCELL, and EPIC. The association between the risk score and the extent of immune cell infiltration was subsequently evaluated. To investigate the disparities in immune cell composition and immune signaling pathways among the risk-defined groups, single-sample gene set enrichment analysis (ssGSEA) was performed using the "gsva" package (version 1.52.3). This analysis calculated the enrichment scores for 16 immune cells and 13 immune signaling pathways. Furthermore, the CIBERSORT [23] algorithm helped enumerate 22 immune cell types' proportions, and subsequently their potential impact on survival was analyzed.

### Tumor mutational burden analysis

TCGA's somatic mutation data were processed with the "maftools" R package (version 2.20.0) [24], which facilitated the generation of waterfall plots. Furthermore, tumor mutational burden (TMB) values were calculated for each OC case, and survival analysis was subsequently performed to elucidate the relationship between TMB values and the prognostic risk score.

### Functional enrichment analysis

Gene Set Enrichment Analysis (GSEA) [25] was employed to probe into the molecular mechanisms underpinning the differences between the risk-defined groups.

### Prediction of drug sensitivity profiles

The half-maximal inhibitory concentration (IC50) is a term used to indicate the concentration of an inhibitor that results in a 50% inhibition of specific biological processes [26]. A smaller IC50 value indicates a higher sensitivity to the drug. The R package "prophetic" (version 0.5) [27] was utilized to predict drug IC50 values using gene expression data. Following that, we compared these values between the high-risk and low-risk groups.

### Cell culture and quantitative real-time PCR

This study employed three different human cell lines, namely A2780, OVCAR3, and HOSEPiC, for the investigation. A2780 and OVCAR3 are human OC cell lines, while HOSEPiC represents the human ovarian surface epithelial cell line. These cell lines were cultured in RPMI 1640 medium (Procell, RPMI-1640), supplemented with 10% fetal bovine serum (FBS), at a temperature of 37 °C and an atmospheric CO_2_ concentration of 5%. For the extraction of total RNA, TransZol Up (TransGen, ET111-01) was utilized, followed by reverse transcription using the HiScript III RT SuperMix (+ gDNA wiper) (Vazyme, R323-01) to generate complementary DNA (cDNA). To quantify the gene expression levels, the quantitative real-time polymerase chain reaction (qRT-PCR) analysis was conducted using ChamQ Universal SYBR qPCR Master Mix (Vazyme, Q711-03). To ensure accurate normalization, the housekeeping gene GAPDH was utilized as an internal control, with gene expression quantified using the 2^−ΔΔCT^ method. PCR primers for HGSNAT, AP2A1, GZMB, LAMP3, CHMP4C, NDUFC2, RAB34, CYBRD1, UNC13D, and FNIP1 used in the construction of the risk signature were purchased from Sangon Biotech (Sangon, China). The primer sequences can be found in Supplementary Table S1.

### Statistical analysis

Statistical analysis was conducted using R software, RStudio, and GraphPad Prism 9.5 software. Differences among groups were compared using ANOVA tests, and between two groups using either Student’s t-test or the Wilcoxon rank-sum test. A significance threshold of p < 0.05 was established to determine statistical significance. Asterisks were used to denote the corresponding p values, with * indicating p < 0.05, ** indicating p < 0.01, *** indicating p < 0.001, and **** indicating p < 0.0001.

## Results

### Functional enrichment analysis and PPI network of LRGs

**Fig. 1:**
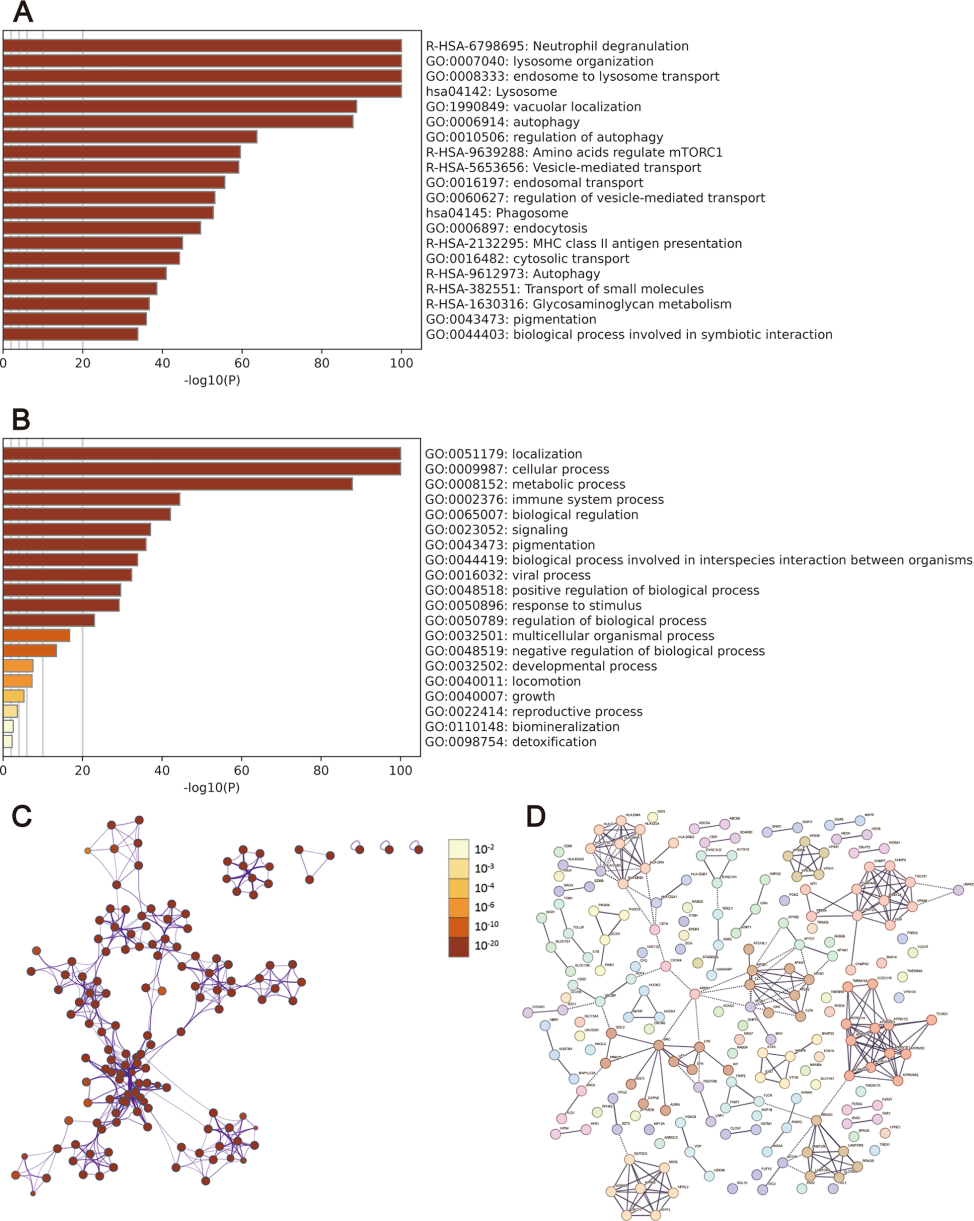
Enrichment analysis was conducted on LRGs, and a PPI network was constructed for these genes. **A** A heatmap displaying enriched GO, KEGG, and Reactome terms. **B** A heatmap showing enriched GO bioprocess terms. **C** A network representation of enriched GO, KEGG, and Reactome terms, color-coded based on their P values. **D** The PPI network showing the interactions of the LRGs

The functional and pathway enrichment of LRGs was analyzed using Metascape to gain deeper insights into their biological functions. The GO enrichment analysis demonstrated significant enrichment of LRGs in various biological processes. These processes include lysosome organization, endosome to lysosome transport, vacuolar localization, autophagy, regulation of autophagy, cellular localization, cellular processes, and metabolic processes. Kyoto Encyclopedia of Genes and Genomes (KEGG) and Reactome pathways analysis underscored connections with neutrophil degranulation, lysosomes, amino acid regulation of mTORC1, and vesicle-mediated transport. Notably, the terms related to lysosomes exhibited the highest enrichment (Fig. 1A–C). To investigate the connections between the LRGs, we constructed a PPI network using data from the STRING database (Fig. 1D).

### Development and validation of a prognostic signature based on lysosomal-related genes

**Fig. 2:**
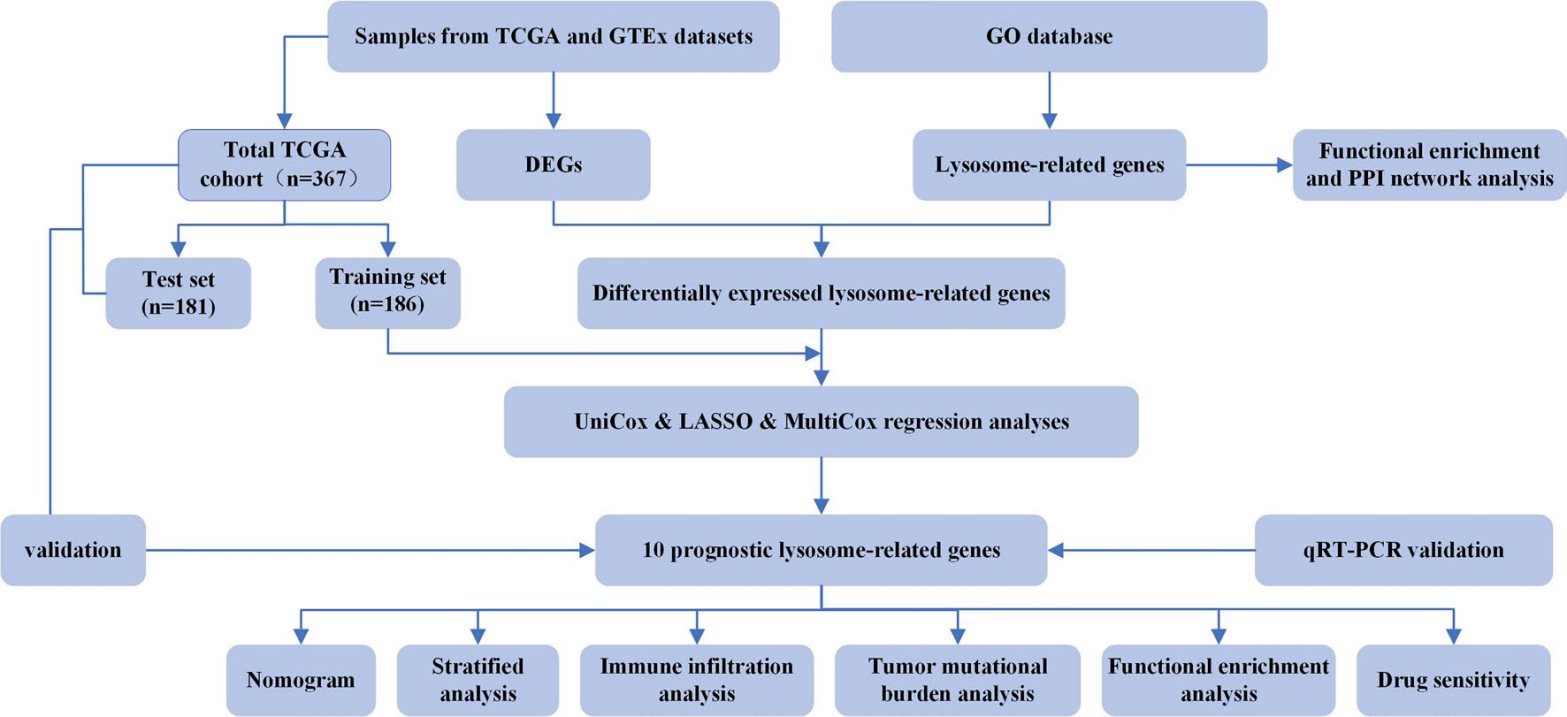
The flowchart of this study

**Fig. 3:**
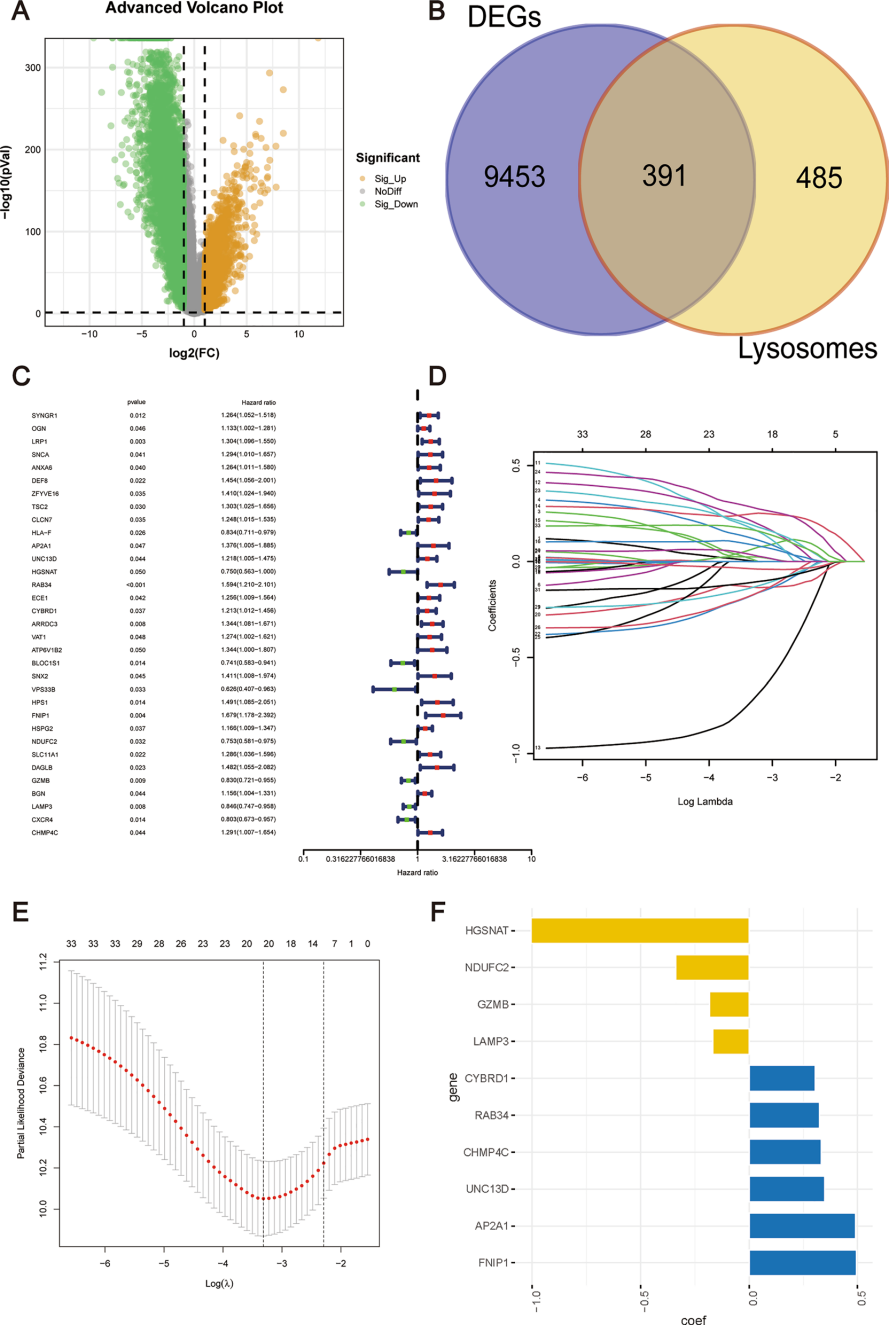
Sequential analysis for the determination of prognostically significant LRGs in OC. **A** Illustration of differential expression analysis comparing OC tissues to normal controls, identifying genes with altered expression. **B** Venn diagram analysis to identify genes that overlap between the DEGs and LRGs. **C** Application of univariate Cox regression to identify LRGs with significant prognostic value. **D** Assessment of the distribution of LASSO coefficients. **E** Selection of the optimal penalty parameter in the LASSO model via cross-validation, ensuring model reliability. **F** Evaluation of the distribution of selected genes and their corresponding regression coefficients

The flowchart of the research is performed in Fig. 2. Following the elimination of the batch effect (Supplementary Fig. 1), a differential analysis was performed, revealing a total of 9844 DEGs when comparing tumor tissues with normal tissues (Fig. 3A). Intersection analysis between the DEGs and LRGs yielded 391 DE-LRGs (Fig. 3B). The TCGA cohort, which comprised n = 367 samples, was randomly segregated into a training set (n = 186) and a test set (n = 181). There were no statistically significant differences observed between the two cohorts in terms of age, staging, and grading (Table 1).

**Table 1:** The clinicopathologic characteristics of the training, test, and the entire TCGA cohort were examined

Covariates	Type	Total	Test	Train	P-value
Age	≤ 65	251 (68.39%)	132 (72.93%)	119 (63.98%)	0.0834
	> 65	116 (31.61%)	49 (27.07%)	67 (36.02%)	
Stage	Stage I	1 (0.27%)	1 (0.55%)	0 (0%)	0.5466
	Stage II	21 (5.72%)	8 (4.42%)	13 (6.99%)	
	Stage III	289 (78.75%)	144 (79.56%)	145 (77.96%)	
	Stage IV	56 (15.26%)	28 (15.47%)	28 (15.05%)	
Grade	G1	1 (0.27%)	1 (0.55%)	0 (0%)	0.5936
	G2	42 (11.44%)	21 (11.6%)	21 (11.29%)	
	G3	324 (88.28%)	159 (87.85%)	165 (88.71%)	

A subsequent univariate Cox regression analysis of DE-LRGs isolated 33 genes with significant prognostic relevance (Fig. 3C). To further refine the gene selection, these genes underwent filtering using LASSO Cox regression analysis (Fig. 3D, E). Using multivariate Cox regression analysis, a set of 10 LRGs was ultimately identified. These genes were then employed to construct the prognostic risk signature (Fig. 3F). The resulting risk scores for patients were computed as follows: risk score = 0.48956*AP2A1 + 0.34752*UNC13D + (− 1.00513*HGSNAT) + 0.32334*RAB34 + 0.30379*CYBRD1 + 0.49377*FNIP1 + (− 0.33839*NDUFC2) + (− 0.18320*GZMB) + (− 0.16683*LAMP3) + 0.33246*CHMP4C.

**Fig. 4:**
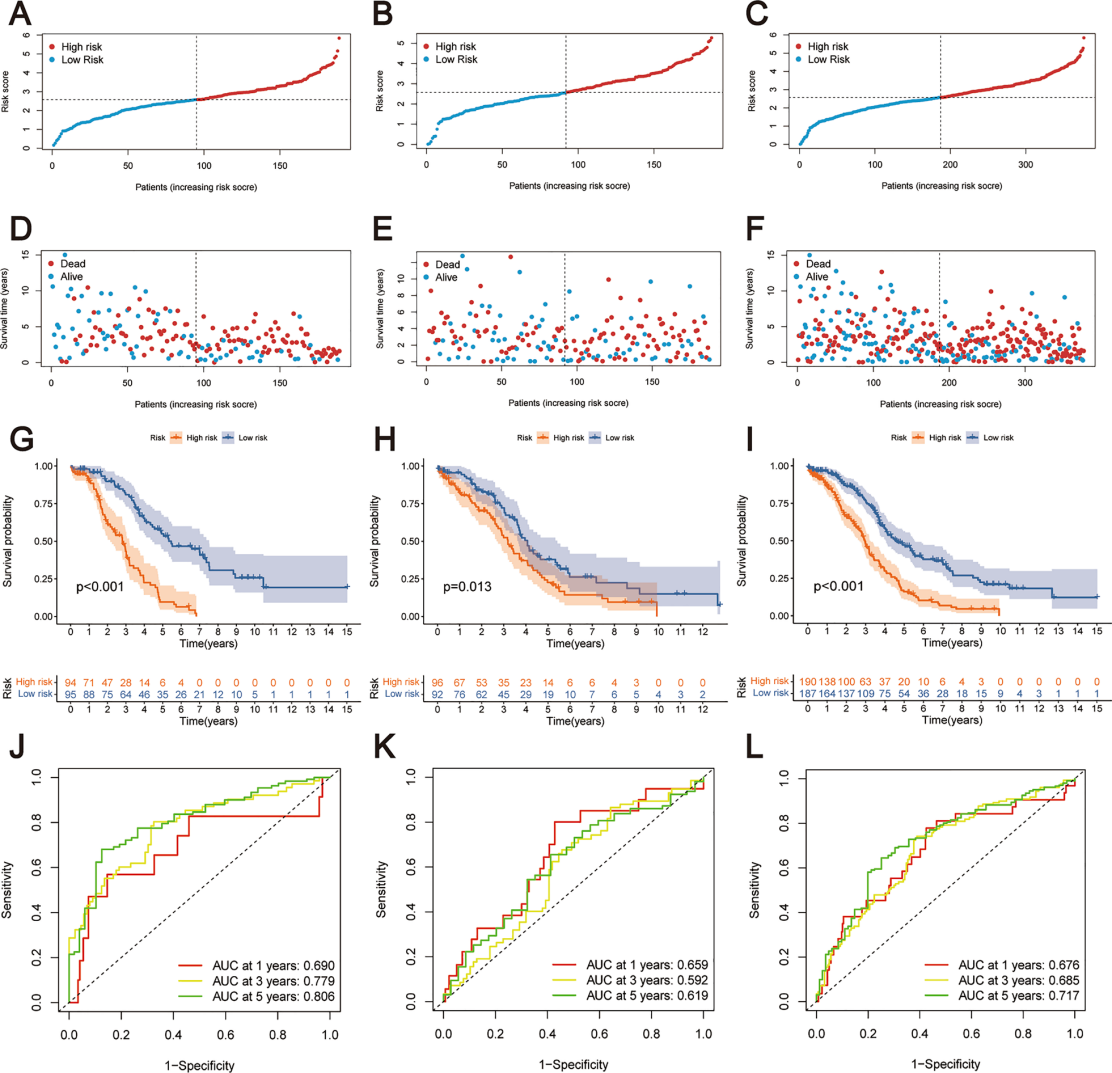
Prognostic model evaluation and validation. **A**–**C** It depicts risk score distributions and their correlation with survival in the training, test, and TCGA cohorts through risk score curves. **D**–**F** Scatter plots were used to visualize the distribution of survival time and risk score in the training, test, and TCGA cohorts. **G**–**I** Kaplan–Meier survival curve analysis was performed in the training, test, and TCGA cohorts. **J**–**L** Additionally, time-dependent ROC curves evaluate the model's accuracy in predicting 1-, 3-, and 5-year overall survival across the training, test, and TCGA patient sets

Based on the median risk score, patients from the training cohort were classified into high-risk and low-risk groups. Notably, there was an observable escalation in mortality rates corresponding to higher risk scores among the patients (Fig. 4A, D). Kaplan–Meier survival curves indicated a statistically significant survival disadvantage in the high-risk group, which corresponded with a lower overall survival rate compared to the low-risk group (Fig. 4G). The ROC analyses affirmed the prognostic model's predictive capacity, with AUC values of 0.690, 0.779, and 0.806 at 1, 3, and 5 years, respectively, demonstrating robust prognostic accuracy (Fig. 4J).

The prognostic utility of the risk score was further validated in the test cohort and the entire TCGA cohort. The categorization of patients into high- and low-risk groups, based on the median risk score, showed that higher risk scores were associated with poorer outcomes. This pattern was consistently replicated across both the test and TCGA cohorts, underscoring the risk score's predictive relevance for patient prognosis.

Furthermore, there was a gradual decrease observed in patient survival time, as illustrated in Fig. 4B, C, E, F. Kaplan–Meier analyses reinforced the finding that patients in the high-risk group had significantly worse prognoses than those in the low-risk group across both the training and test cohorts (Fig. 4H, I). ROC analysis provided AUC metrics of 0.659, 0.592, and 0.619 for 1-, 3-, and 5-year overall survival predictions in the test cohort, respectively (Fig. 4K). For the TCGA cohort, the AUC values for 1-, 3-, and 5-year overall survival were 0.676, 0.685, and 0.717, respectively (Fig. 4L). These findings collectively demonstrate the considerable predictive capability of the risk score in terms of the prognosis of OC patients.

Additionally, to examine the distribution of LRGs, principal components analysis (PCA) was performed, as the complete gene set, and the prognostic model containing 10 lysosomal genes, as seen in Supplementary Fig. 2. The analysis revealed that the 10-lysosomal gene set successfully classifies OC patients into two independent groups, highlighting the high discriminatory capability of the model.

### Independent prognostic analysis was performed, and a stratified analysis of the prognostic risk score was conducted

**Fig. 5:**
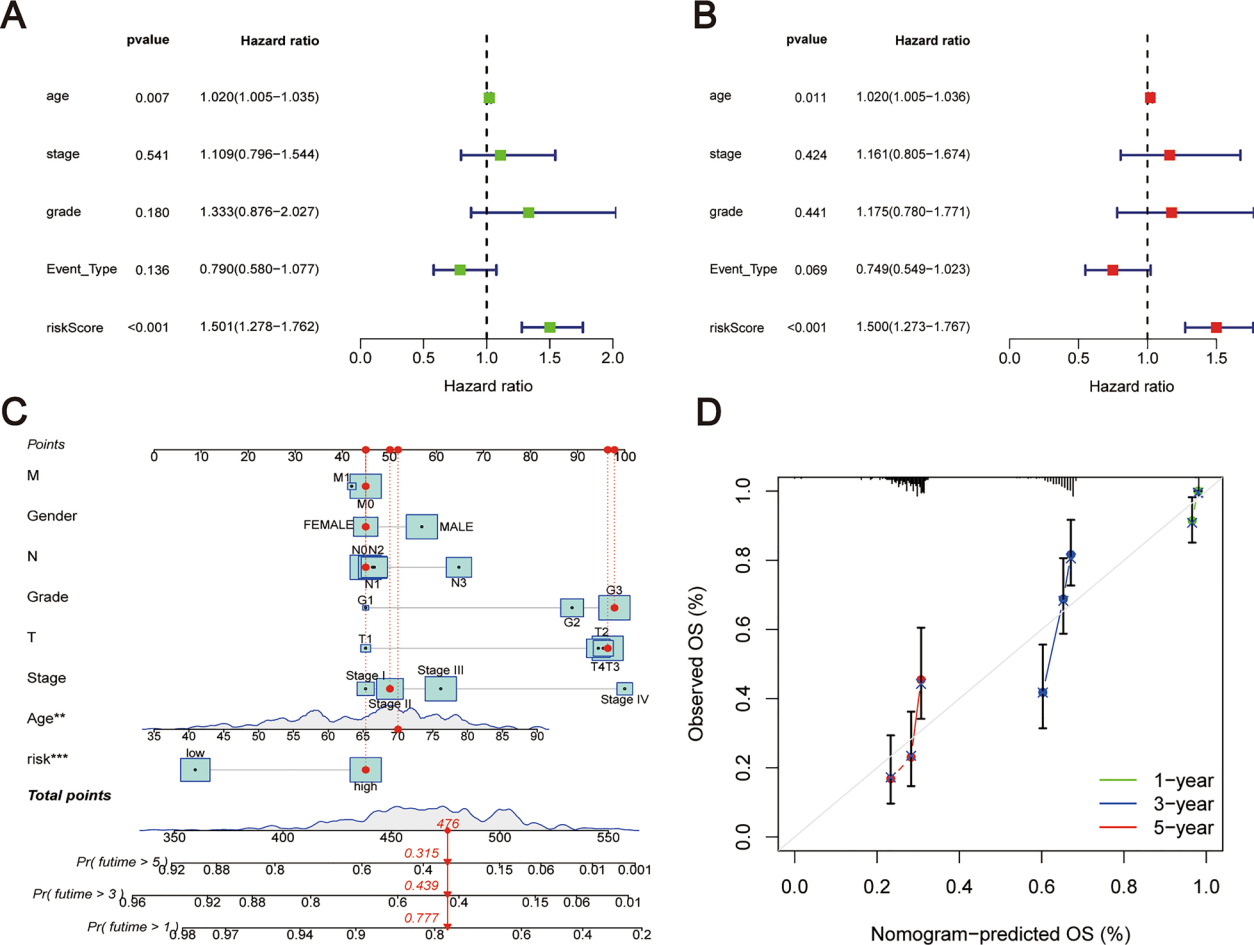
Independent prognostic analyses were conducted to evaluate the risk signature. **A** Univariate Cox regression analysis determining the influence of various clinical factors, including the risk score, on patient prognosis. **B** Multivariate Cox regression analysis further assessing the risk score's prognostic significance alongside other clinical factors. **C** The construction of a nomogram incorporating clinical parameters and the risk score, designed to predict 1-, 3-, and 5-year overall survival for ovarian cancer patients. **D** Calibration curves for the nomogram, indicating the accuracy of survival predictions compared to actual outcomes

In the independent prognostic analysis, both univariate and multivariate Cox proportional hazards regression models were applied to evaluate the standalone predictive value of the risk score. These analyses established that the risk score was significantly associated with the prognosis of OC patients, as delineated in Fig. 5A, B. To improve the prognostic prediction for patients with OC, we constructed a nomogram integrating age, stage, grade, and risk score to forecast 1-, 3-, and 5-year overall survival (OS) rates (Fig. 5C). The predictive reliability of the nomogram was confirmed by calibration curves, which demonstrated a high degree of accuracy in OS predictions for OC patients (Fig. 5D).

**Fig. 6:**
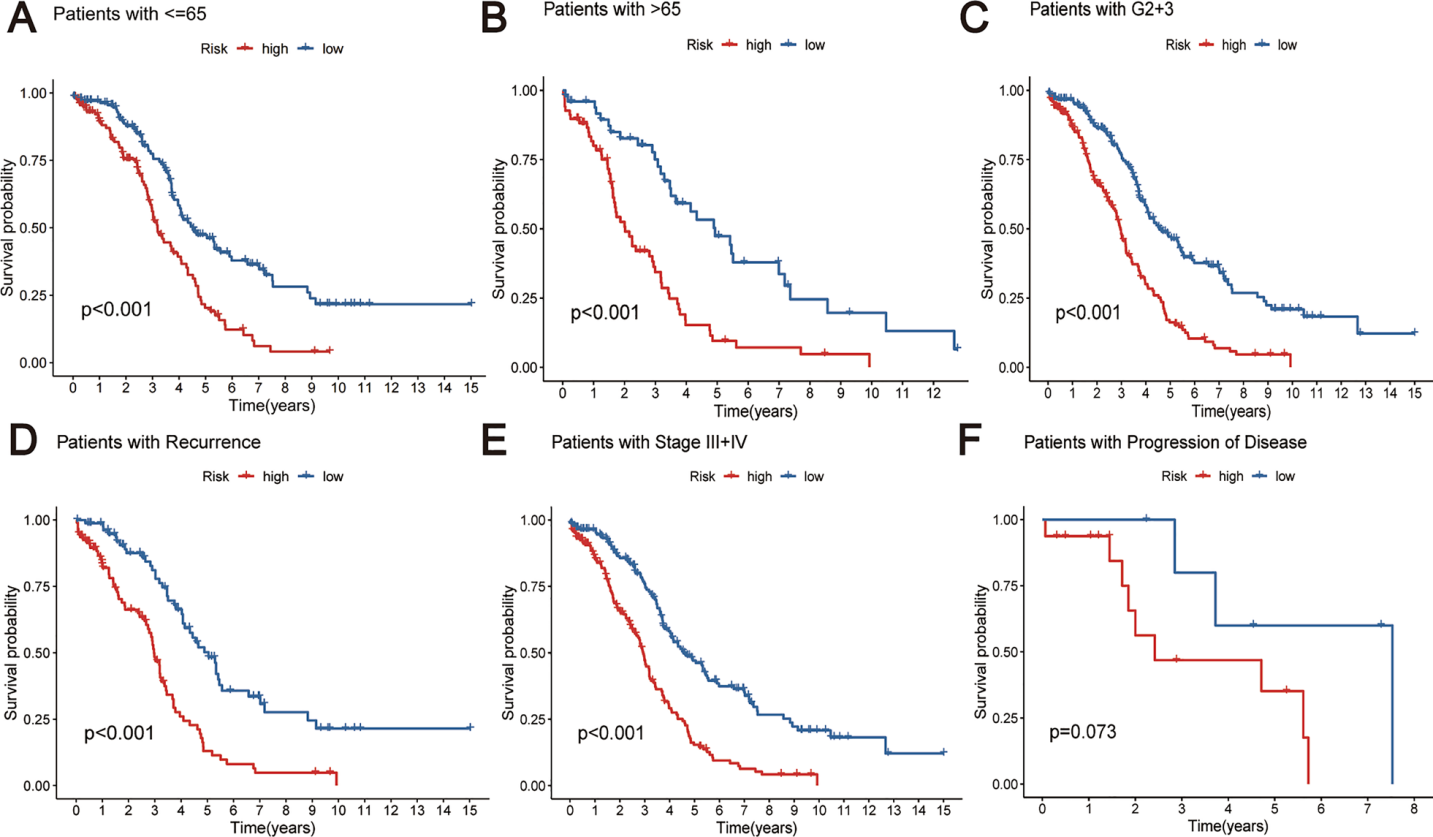
By conducting Kaplan–Meier survival analysis, individuals were stratified based on various clinical characteristics, resulting in the following subgroups: **A** Patients aged ≤ 65 years, **B** Age > 65 years, **C** G2 + 3, **D** Patients with recurrence, **E** Stage III + IV, and **F** Patients with disease progression

Afterward, stratified analyses were performed to investigate the association of the risk score with diverse clinical parameters. In patients stratified by age > 65 years, age ≤ 65 years, differentiation grade G2 + G3, tumor stage III + IV, and presence of recurrence, patients in the high-risk category consistently demonstrated significantly reduced OS when compared to their low-risk counterparts (Fig. 6A–E). Nevertheless, in the subset of patients with progressive disease, no appreciable difference in OS was observed between high and low-risk groups (Fig. 6F). These results underscore the robustness of the risk score as a predictive tool across a spectrum of clinical scenarios in ovarian cancer.

### Immune infiltration analysis

**Fig. 7:**
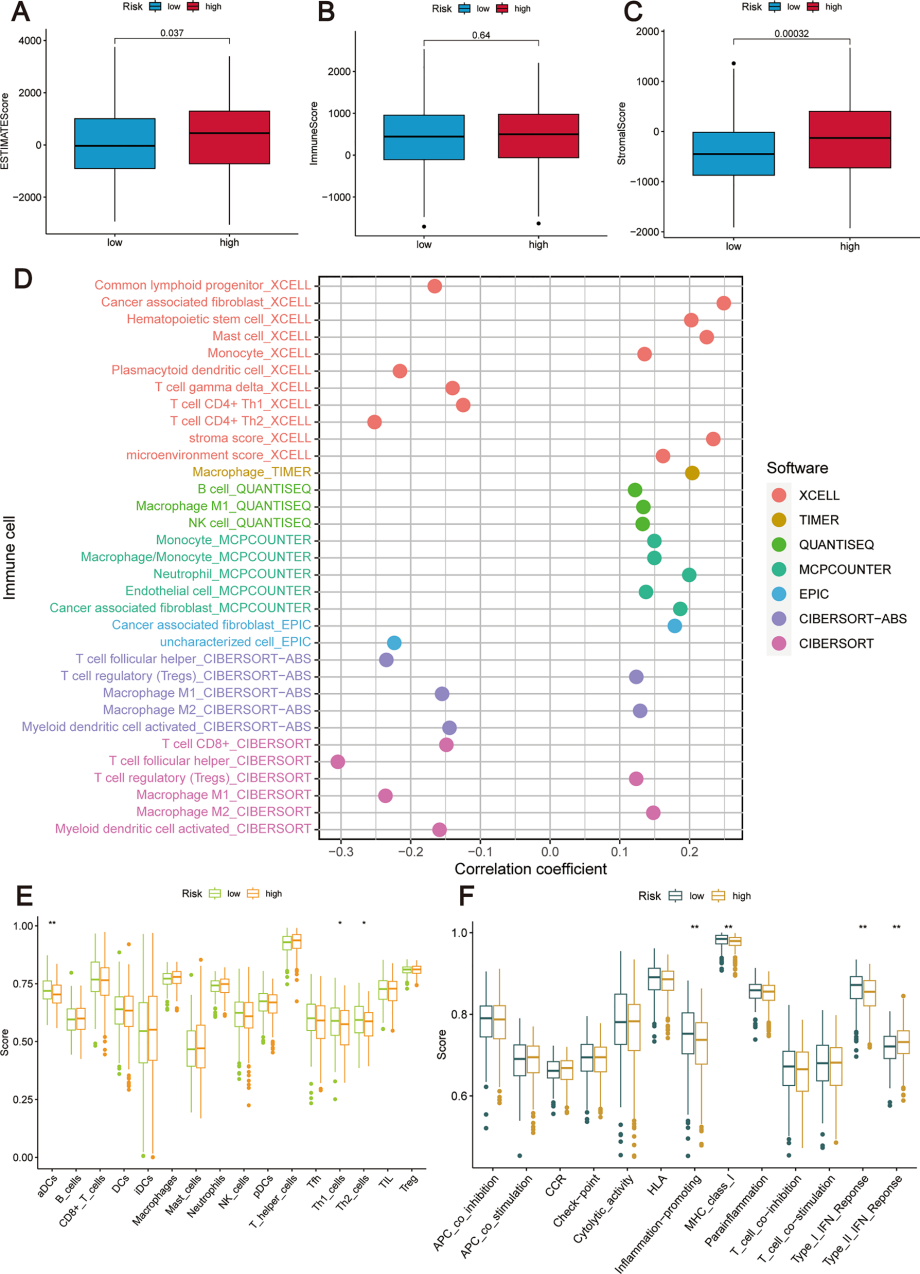
By conducting an immune infiltration analysis, a comparison was made between the high-risk and low-risk groups, resulting in the following outcomes: **A**–**C** The ESTIMATE score, immune score, and stromal score were subjected to a comparative analysis. **D** Correlation analysis illustrating the association between the prognostic risk score and the levels of immune cell infiltration. **E** A comparison was made between the two risk groups regarding the ssGSEA scores of immune cells. **F** The ssGSEA scores of immune function were compared between the two risk groups

In our investigation into the interplay between the risk score and the tumor immune microenvironment, we employed the ESTIMATE algorithm to compute ESTIMATE, immune, and stromal scores for tumor samples. This analysis indicated that the high-risk group was characterized by significantly higher stromal and ESTIMATE scores than the low-risk group, while immune scores did not differ significantly between the groups (Fig. 7A–C). Further correlation analysis assessed the link between the risk score and immune cell infiltration, identifying specific immune cells positively associated with the risk score (Fig. 7D).

A detailed comparison of immune cells and immune signaling pathways between the high-risk and low-risk groups revealed distinct profiles. The low-risk group showed an abundance of activated dendritic cells (aDCs), T helper type 1 (Th1) cells, Th2 cells, and elevated levels of factors promoting inflammation, major histocompatibility complex (MHC) class I, and type I interferon (IFN) response. In contrast, the high-risk group was associated with a heightened type II IFN response (Fig. 7E, F), suggesting a nuanced and differential immune landscape within the tumor microenvironment based on the risk stratification.

**Fig. 8:**
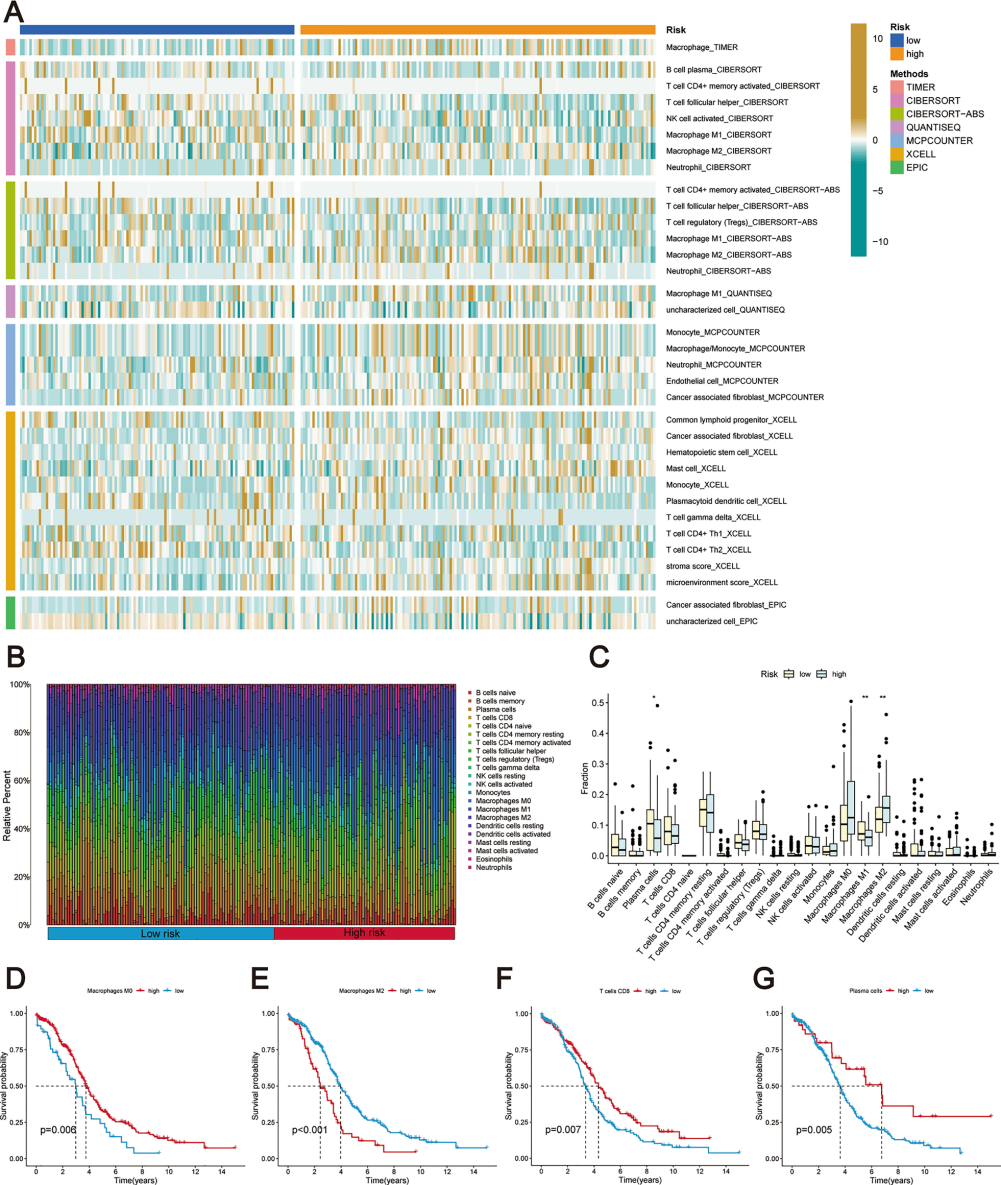
Comprehensive analysis of immune infiltration in relation to prognostic risk score. **A** A heatmap was generated to illustrate the immune infiltration levels based on multiple algorithms. **B** The abundance distribution of 22 immune cell types in both the two risk groups was examined. **C** Box line plots display the relative abundances of these immune cells, visually contrasting the immune landscapes of the two risk groups. **D**–**G** To assess the effect of high and low expression levels of specific immune cells on patient survival rates, Kaplan–Meier survival curves were generated. Specifically, this analysis investigated the impact of (**D**) M0 macrophages, (**E**) M2 macrophages, (**F**) CD8 + T cells, and (**G**) Plasma cells

Figure 8A illustrates the utilization of multiple algorithms including TIMER, CIBERSORT, CIBERSORT-ABS, QUANTISEQ, MCP-COUNTER, XCELL, and EPIC to investigate the variation in immune infiltration levels between the high-risk and low-risk groups. By employing the CIBERSORT algorithm, we quantified 22 distinct immune cell types, uncovering significant disparities in immune composition between risk-defined groups (Fig. 8B, C). Notably, plasma cells and M1 macrophages were more prevalent in the low-risk group, while M2 macrophages were predominant in the high-risk group.

Furthermore, we conducted survival analyses to examine the impact of immune cell enrichment on patient prognosis. Based on our findings, patients with higher infiltration levels of M0 macrophages, CD8 + T cells, and plasma cells exhibited significantly better prognoses compared to those with lower levels of infiltration. Conversely, patients with higher levels of M2 macrophage infiltration demonstrated poorer prognostic outcomes, as depicted in Fig. 8D–G. In conclusion, the collective results emphasize the association between the prognostic risk score and TME, underscoring its potential significance in predicting patient outcomes.

### Tumor mutational burden analysis performed

**Fig. 9:**
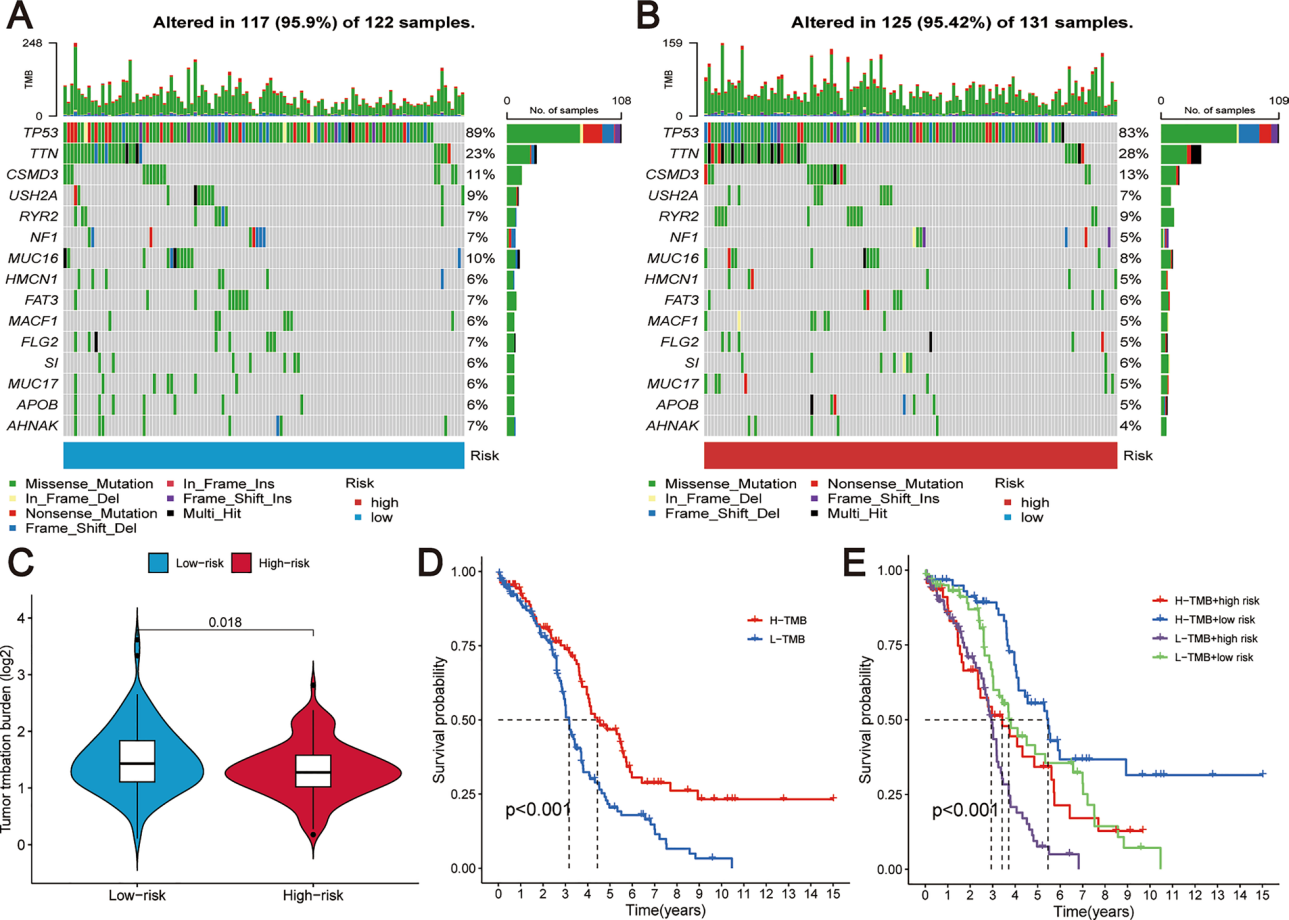
Correlation between TMB, risk score, and survival in OC. **A** Waterfall plot of the genetic mutation profile in the low-risk patient group. **B** An illustration of the mutation distribution observed in the high-risk group is represented in the form of a waterfall plot. **C** Analysis of the association between the prognostic risk score and TMB. **D** Kaplan–Meier survival curves for patient groups classified by high and low TMB, demonstrating the impact of TMB on overall survival. **E** Survival analysis based on both TMB and risk score as combined prognostic factors

The analysis of gene mutation profiling revealed that mutations were present in 95.9% (117 out of 122 samples) of the low-risk group and 95.42% (125 out of 131 samples) of the high-risk group, with a marginally greater mutation frequency in the low-risk group. The most frequently mutated genes across both cohorts were TP53, TTN, and CSMD3, predominantly featuring missense mutations (Fig. 9A, B). Furthermore, TMB was calculated for each patient sample. Interestingly, the low-risk group displayed higher TMB values compared to the high-risk group (Fig. 9C). Subsequently, dividing the OC samples based on median TMB values into high and low TMB groups facilitated a survival analysis. This analysis showed that patients with high TMB had significantly better OS compared to those with low TMB (Fig. 9D). A nuanced survival analysis considering both TMB and risk score revealed that patients with high TMB and low-risk scores had the most favorable OS, whereas those with low TMB and high-risk scores had the least favorable outcomes (Fig. 9E). These findings highlight a complex interplay between TMB, risk score, and patient survival, and suggest that TMB could serve as a complementary prognostic factor alongside the risk score in OC.

### Functional enrichment analysis

**Fig. 10:**
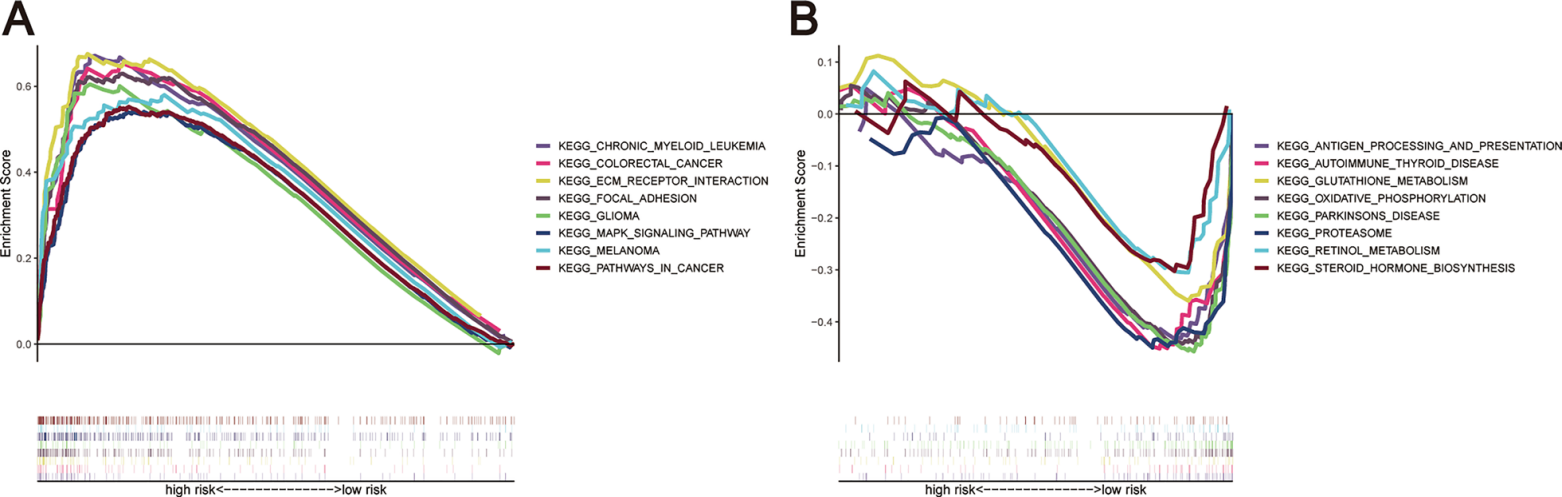
The GSEA was conducted on both the high and low-risk groups, with the following outcomes: **A** GSEA analysis of the high-risk group. **B** GSEA analysis of the low-risk group

We utilized GSEA to investigate the molecular mechanisms underlying the prognostic signature. The analysis uncovered significant enrichment of various pathways in the high-risk group, such as chronic myeloid leukemia, colorectal cancer, ECM-receptor interaction, focal adhesion, glioma, MAPK signaling pathway, melanoma, and pathways in cancer. In contrast, the low-risk group displayed pathway enrichments, including antigen processing and presentation, autoimmune thyroid disease, glutathione metabolism, oxidative phosphorylation, Parkinson's disease, proteasome, retinol metabolism, and steroid hormone biosynthesis, as illustrated in Fig. 10A, B.

### Drug sensitivity prediction

**Fig. 11:**
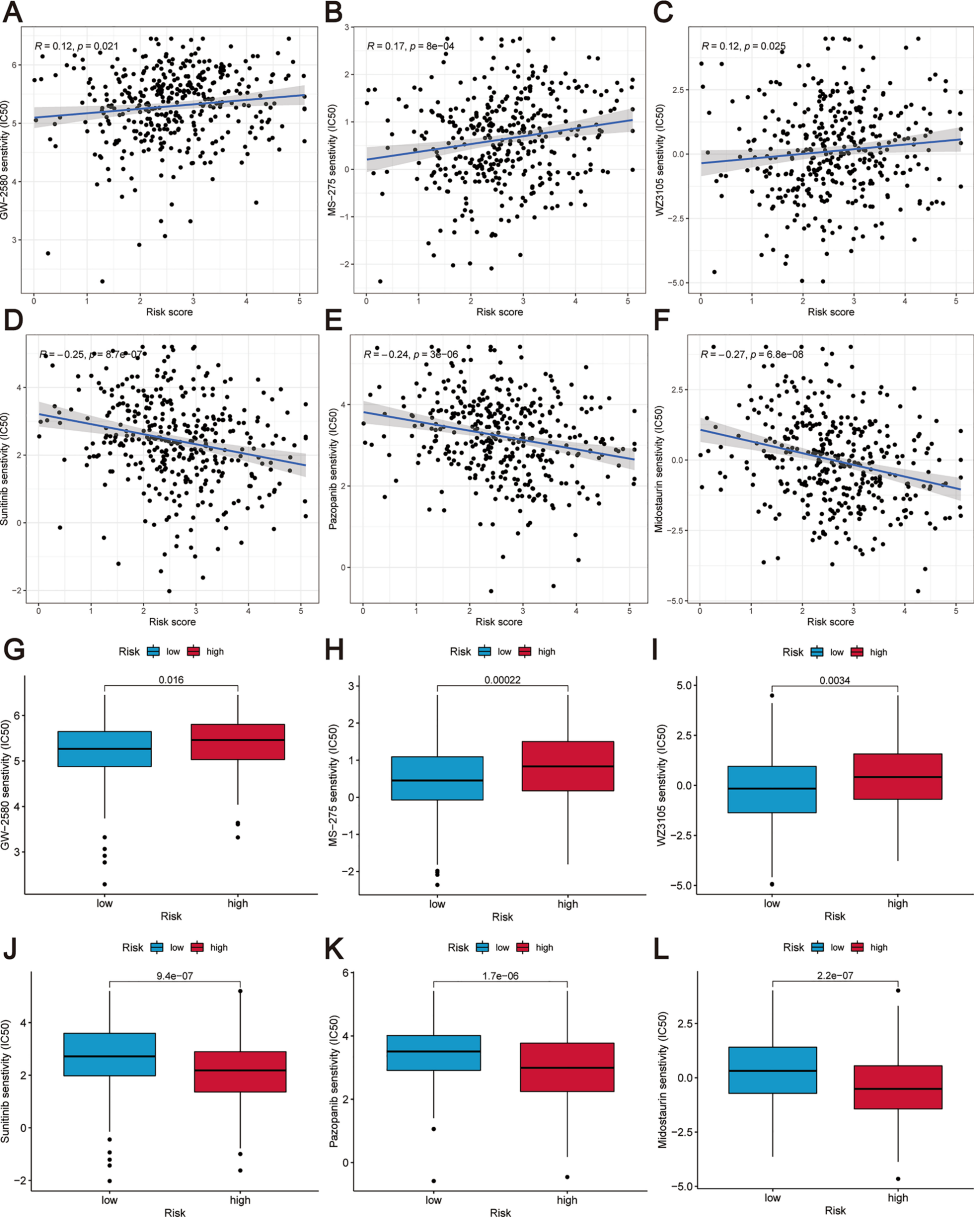
The correlation between the risk score and therapeutic drugs is illustrated as follows: **A**–**F** The relationship between risk scores and drug IC50 values is demonstrated for the following compounds: **A** GW-2580, **B** MS-275, **C** WZ3105, **D** sunitinib, **E** pazopanib, and **F** midostaurin. Moreover, the variations in drug IC50 values between the high and low-risk groups are depicted for (**G**) GW-2580, (**H**) MS-275, (**I**) WZ3105, (**J**) sunitinib, (**K**) pazopanib, and (**L**) midostaurin

In our study, we investigated the relationship between the risk score and drug sensitivity by comparing the IC50 values of various drugs in the high-risk and low-risk groups. The analysis demonstrated a positive correlation between the risk scores and the IC50 values of GW-2580, MS-275, and WZ3105, as shown in Fig. 11A–C. Moreover, we observed that the IC50 values of GW-2580, MS-275, and WZ3105 consistently showed lower values in the low-risk group than in the high-risk group, as depicted in Fig. 11G–I. These results imply the potential effectiveness of these drugs in patients categorized as low-risk. Conversely, the IC50 values of sunitinib, pazopanib, and midostaurin showed a negative correlation with the risk scores, as illustrated in Fig. 11D–F. Furthermore, there was a notable decrease in the IC50 values of these drugs in the high-risk group, suggesting heightened sensitivity among patients in this group, as depicted in Fig. 11J–L. These findings suggest that the risk score may inform the selection of therapeutic agents, with certain drugs being potentially more effective in high-risk patients.

### Validation of 10-gene prognostic signature

**Fig. 12:**
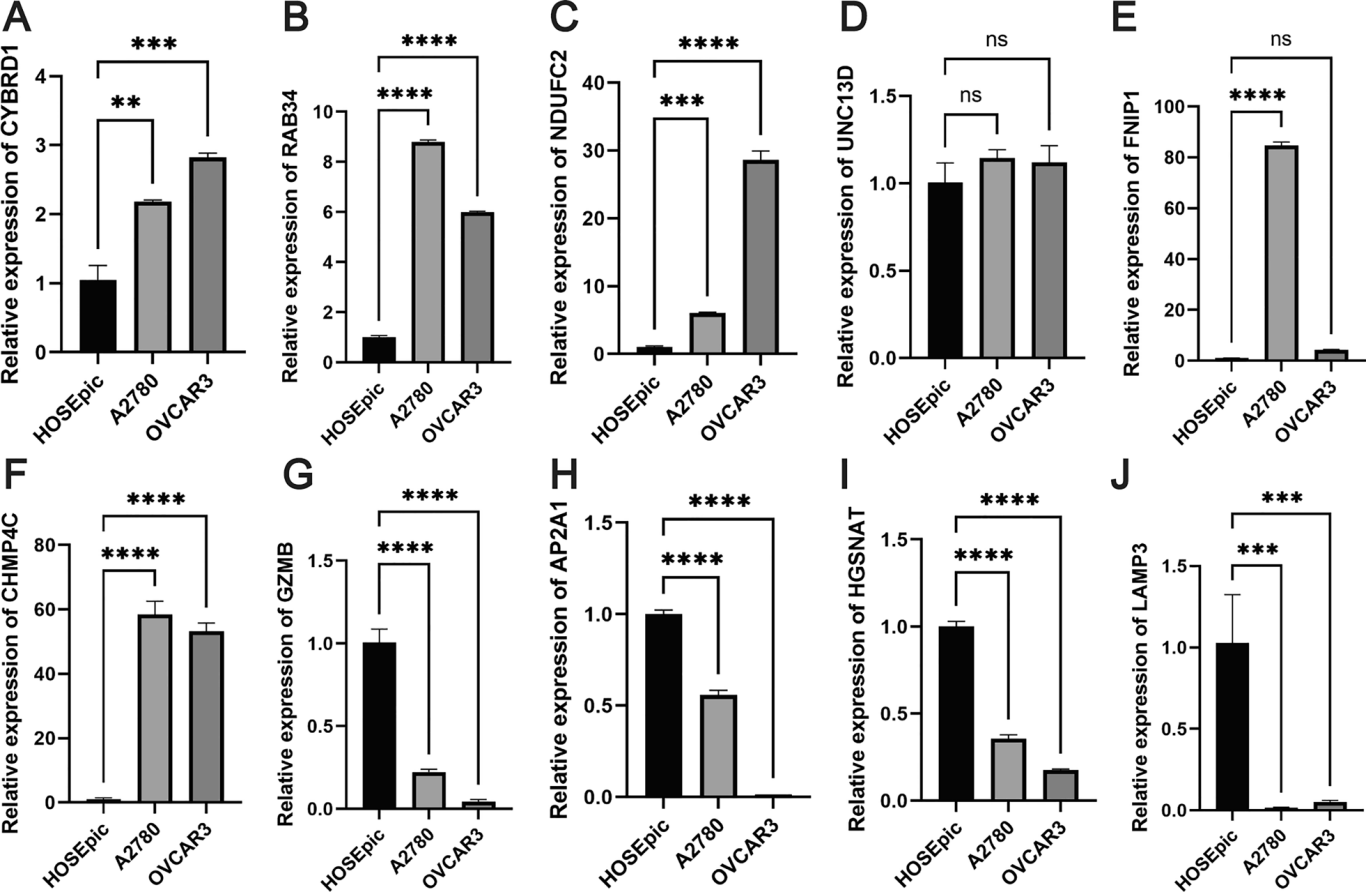
The expression levels of 10 key LRGs, denoted by their symbols, in normal ovarian surface epithelial cells (HOSEpiC) and ovarian cancer cells (A2780 and OVCAR3) are shown below: **A** CYBRD1, **B** RAB34, **C** NDUFC2, **D** UNC13D, **E** FNIP1, **F** CHMP4C, **G** GZMB, **H** AP2A1, **I** HGSNAT, and **J** LAMP3

In the experimental validation of the 10-gene prognostic signature, qRT-PCR analyses were performed on OC cell lines and compared with normal ovarian surface epithelial cells. Our findings demonstrated a down-regulation of the HGSNAT, AP2A1, GZMB, and LAMP3 genes in the OC cell lines, In contrast, the OC cell lines exhibited up-regulation of CHMP4C, NDUFC2, RAB34, and CYBRD1 expression levels. Furthermore, UNC13D and FNIP1 also showed increased expression levels in OC cells, albeit without statistical significance (Fig. 12).

## Discussion

OC continues to be a formidable malignancy, severely impacting women's health globally [28]. The identification of effective biomarkers for prognostic prediction in OC remains a key focus of current research. Lysosomal structural and functional abnormalities have been extensively documented to contribute significantly to tumor pathogenesis. Consequently, therapeutics targeting lysosomal mechanisms have emerged as a potential strategy in cancer treatment. Despite prior research on lysosomes, their specific role in predicting outcomes for OC is still not fully understood. Consequently, the development of a prognostic signature based on LRG genes is urgently needed to accurately predict the prognosis of OC patients.

In our research, we undertook a comparative evaluation of DEGs from both the TCGA and GTEx repositories. We then utilized a spectrum of statistical methods, such as univariate, LASSO, and multivariate Cox regression analyses, to hone in on a set of 10 LRG genes. The intention behind this was to devise a prognostic marker that could accurately forecast the outcome for OC patients. Our investigation pinpointed HGSNAT, NDUFC2, GZMB, and LAMP3 as safeguarding elements, whereas AP2A1, UNC13D, RAB34, CYBRD1, FNIP1, and CHMP4C emerged as hazardous indicators. Within the training group, a rise in risk values correlated with unfavorable OS results. Moreover, this risk metric underwent validation in both the test sample and the comprehensive TCGA sample, underscoring its robust forecasting prowess. In essence, both univariate and multivariate Cox regression evaluations ascertained the risk index as a standalone predictor for OC prognosis. Additionally, a tailored nomogram was crafted to anticipate the life expectancy of ovarian cancer patients, incorporating variables like risk indices, age, tumor grade, and stage. The efficacy of this nomogram in estimating OS was gauged through calibration curve techniques. Moreover, an exhaustive stratified examination was conducted to understand the interplay between the risk index and various clinical features. Significantly, our results showcased that the risk metric remained a consistent and accurate tool for predicting outcomes in patients exhibiting stage III/IV conditions, grade II/III malignancies, recurrent OC episodes, and within age brackets both exceeding and up to 65 years.

Extant research has illuminated the roles of particular genes as they pertain to OC. Notably, Granzyme B (GZMB), a serine protease, is excreted by entities such as natural killer (NK) cells and cytotoxic T lymphocytes (CTLs) [29]. GZMB stands as a defensive element in OC, with elevated levels heralding an optimistic outcome for patients, underpinned largely by its pivotal influence on immune cellular dynamics[30, 31]. Transitioning to the lysosomal-associated membrane protein 3 (LAMP3), this protein hails from the lysosomal-associated membrane protein lineage. Its role is indispensable in preserving the lysosomal membrane's structural fortitude. Strikingly, when comparing mRNA and protein expressions of LAMP3, levels were discernibly augmented in epithelial ovarian cancer (EOC) relative to benign tissue counterparts. This heightened LAMP3 expression bore correlations with less promising patient outcomes, suggesting its utility both diagnostically and therapeutically for EOC [32].

Diving into Jiao's investigations, APA21 emerged as an OC risk determinant during their prognostic signature formulation [33]. In parallel, another research initiative underscored that a signature enriched with AP2A1 bore potential diagnostic merit for epithelial ovarian cancer [34]. Delving into the RAB34 protein, an integral member of the RAB protein cohort, it predominantly resides within the Golgi apparatus, playing a cardinal role in processes such as giant cytosolic absorption and intricate protein translocation [35]. Intriguingly, RAB34 exhibits the aptitude to ally with the RAB-interacting lysosomal protein (RILP), a key entity in directing lysosomes toward the perinuclear vicinity, thereby modulating their spatial orientation [36]. There's a scholarly accord that in specific paclitaxel-resistant serous ovarian cancer samples, miR-9 expression was diminished, and intriguingly, RAB34 was pinpointed as its direct influencer [37]. This raises the possibility of RAB34 playing a part in the inherent resistance mechanism of this ovarian cancer variant. However, unpacking the multifaceted dynamics between RAB34 and OC's trajectory is paramount.

The enzyme cytochrome b reductase 1 (CYBRD1), endowed with specific reductase capabilities, facilitates the Fe^3+^ to Fe^2+^ transition, thereby acting as a sentinel in ferrous iron assimilation processes [38]. Comprehensive meta-evaluations highlighted that a surge in CYBRD1 expression correlated with a bleaker prognosis for OC afflicted patients [39]. A study helmed by Chen and collaborators [40] underscored that CYBRD1, potentially indicative of ovarian serous cystadenocarcinoma (OV), witnessed pronounced elevations in OV, with its surges linked to factors like lymphatic dissemination, disease progression, and clinical trajectories less favorable. The contemporary research milieu indicates a spike in CHMP4C expression in OC specimens, positioning it as a likely diagnostic and therapeutic pivot for OC [41]. Concurrently, CHMP4C has been tagged as a gene amplifying OC susceptibility[42, 43]. Yet, the contributions of HGSNAT, NDUFC2, UNC13D, and FNIP1 within the OC spectrum remain nebulous. We performed an external evaluation using qRT-PCR to validate the gene expression patterns of 10 LRGs in OC cells. However, the results for a few genes differed from the insights of previous studies, e.g., AP2A1. These discrepancies could be attributed to the fact that these genes were validated only at the RNA level, the small number of OC cell types selected, or the multifaceted tumor nuances inherent in OC, which emphasizes the imperative for us to follow up with more extensive studies for validation.

Historically, TME encompasses a variety of elements, from immune cells, stromal cells, and fibroblasts to the extracellular matrix. This complex milieu has increasingly been recognized as pivotal in the onset and advancement of OC, garnering more focused academic scrutiny [44]. In our exploratory study, we discerned marked disparities in the distribution of immune cells across the two risk categories. To be specific, the group categorized as lower risk manifested a pronounced surge in aDCs, Th1 cells, Th2 cells, plasma cells, and M1 macrophages. Conversely, the group marked as higher risk predominantly displayed an abundance of M2 macrophages. Delving deeper through the utilization of the ssGSEA technique, it became apparent that the immune functionality was considerably more vigorous in the low-risk category in contrast to its high-risk counterpart. Moreover, an escalated presence of M2 macrophages was linked to less favorable patient outcomes. In contrast, a heightened presence of M0 macrophages, CD8 + T cells, and plasma cells was decidedly associated with more promising results.

The role of aDCs in antigen presentation and subsequent immune response modulation is well-documented [45, 46]. Th1 and Th2 cells represent subsets of CD4 + T cells, each distinguished by their unique cytokine secretions. Th1 cells are known to release pro-inflammatory agents such as IFN-γ and interleukin-2 (IL-2), vital players in directing cellular immune reactions. On the other hand, Th2 cells emit anti-inflammatory agents, like IL-10, which are instrumental in guiding humoral immunity [47]. A skewed balance between Th1 and Th2 cells has been linked to OC development and prognosis [48]. The imperative part played by CD8 + T cells in countering tumor growth is widely recognized[49]. Within the OC landscape, abundant infiltration of CD8 + T cells often signifies a promising outlook [50, 51]. The implications of plasma cells within OC, however, remain a point of contention. As per Lundgren et al. [52], the heightened presence of both B cells and plasma cells suggests a less-than-favorable trajectory for OC. In contrast, Kroeger et al. [53] posited a positive relationship between plasma cell infiltration and CD8 + T cells, tertiary lymphoid formations, and encouraging outcomes, especially in cases marked by high-grade serous ovarian cancer (HGSC).

Our research has decisively shown that OC patients with elevated plasma cell infiltration tend to experience a markedly better prognosis. As a result, it's essential to pursue further studies to uncover the precise mechanisms by which plasma cells influence OC. Contemporary studies reinforce the understanding that M0 macrophages can evolve into one of two specific phenotypes: the classically activated macrophages (M1) and the alternatively activated macrophages (M2) [54–56]. Characteristically, M1 macrophages release pro-inflammatory agents like TNF, IL-1, IL-6, and IL-12, which possess tumor-fighting properties. In contrast, M2 macrophages curtail the secretion of pro-inflammatory agents and instead release anti-inflammatory cytokines, playing a role in fostering tumor development [57, 58]. Importantly, within the context of OC, a pronounced infiltration of M1 macrophages correlates with a positive prognosis. However, a heightened presence of M2 macrophages indicates a less optimistic outcome and aids in the advancement of OC via immune inhibitory pathways [59]. These insights align closely with the conclusions drawn from our research.

TMB is a measure of the number of mutations within a tumor and has been recognized as a potential biomarker for predicting the efficacy of immunotherapy treatments [60, 61]. Remarkably, a plethora of research has showcased that individuals with an elevated TMB often experience enhanced outcomes from immunotherapy [62, 63]. Such outcomes are likely due to the augmented generation of neoantigens in tumors that have a higher TMB. This surge aids the immune system in better-identifying tumor cells, subsequently leading to a more optimistic prognosis for the affected individuals [64, 65].

During the course of our research, we noted that those allocated to the low-risk category displayed a pronounced TMB in contrast to their counterparts in the high-risk bracket. This observation intimates that individuals in the low-risk cluster potentially respond more positively to immunotherapeutic interventions. Moreover, our findings confirmed that OC patients with higher TMB had significantly better overall survival, and when combining TMB with risk scores, patients with high TMB and low-risk scores had the most favorable prognoses. This underscores the enhanced precision in prognosis prediction when mutation metrics and risk evaluations are jointly considered. Intriguingly, the gene TP53, predominantly marked by missense mutations, had the most frequent mutation occurrence in both risk spectrums. Recognized as a tumor inhibitor gene, TP53 plays a pivotal role in orchestrating a spectrum of cellular activities like regulating cell cycle, DNA restitution, programmed cell death, and metabolic processes [66, 67]. TP53 mutations are almost ubiquitous across various tumor types and are strongly associated with a poorer prognosis for patients[68, 69].

The GSEA demonstrated a significant association between the identified prognostic signature and key pathways involved in immunity, metabolism, and cancer, including antigen processing and presentation, glutathione metabolism, pathways in cancer, and MAPK signaling pathway. The results imply that the prognostic marker could hold significant implications for the biological understanding of OC. Furthermore, to enhance treatment strategies for patients, we utilized risk scores to predict potentially effective drugs for OC. The data analysis indicates that those categorized under the low-risk bracket might find a therapeutic advantage with GW-2580, MS-275, and WZ3105. Conversely, those ascribed to the high-risk category might respond well to treatments with sunitinib, pazopanib, and midostaurin. Of note, sunitinib and pazopanib are small-molecule ATP-competitive tyrosine kinase inhibitors, which have shown promise in the treatment of OC. However, the implementation of specific protocols for these drugs requires further study and investigation [70].

Nevertheless, it is imperative to acknowledge a few constraints within this study. Primarily, its retrospective nature that relied on publicly available databases warrants further confirmation through prospective multicenter data. Moreover, a more comprehensive understanding of the roles of LRGs in OC will require additional experimental investigations to confirm our bioinformatics findings and to further elucidate the molecular mechanisms at play.

## Conclusions

Within the confines of the present investigation, an exclusive prognostic model integrating ten LRGs has been meticulously devised and subsequently verified as an autonomous prognostic determinant in forecasting OS rates among individuals afflicted with OC. Additionally, this research has adeptly shed light on the nuanced relationship between the prognostic framework, the TME, and mutation patterns, effectively harnessing this model to predict both therapeutic efficacy and patient-specific treatment responses. Consequently, this innovative approach offers considerable potential in facilitating tailor-made patient care strategies.

## Supplementary Information

Supplementary Material 1.

Supplementary Material 2.

## Data Availability

The datasets used in this study are all publicly available. These datasets can be obtained from the TCGA (https://portal.gdc.cancer.gov) and the GO consortium (http://geneontology.org/).

## References

[1] Siegel RL, Miller KD, Wagle NS, et al. Cancer statistics, 2023. CA Cancer J Clin. 2023;73(1):17–48. 36633525 10.3322/caac.21763

[2] Lheureux S, Braunstein M, Oza AM. Epithelial ovarian cancer: evolution of management in the era of precision medicine. CA Cancer J Clin. 2019;69(4):280–304. 31099893 10.3322/caac.21559

[3] Stewart C, Ralyea C, Lockwood S. Ovarian cancer: an integrated review. Semin Oncol Nurs. 2019;35(2):151–6. 30867104 10.1016/j.soncn.2019.02.001

[4] Settembre C, Fraldi A, Medina DL, Ballabio A. Signals for the lysosome: a control center for cellular clearance and energy metabolism. Nat Rev Mol Cell Biol. 2013;14(5):283–96. 23609508 10.1038/nrm3565 PMC4387238

[5] Appelqvist H, Wäster P, Kågedal K, Öllinger K. The lysosome: from waste bag to potential therapeutic target. J Mol Cell Biol. 2013;5(4):214–26. 23918283 10.1093/jmcb/mjt022

[6] Lamming DW, Bar-Peled L. Lysosome: the metabolic signaling hub. Traffic. 2019;20(1):27–38. 30306667 10.1111/tra.12617 PMC6294686

[7] Lim CY, Zoncu R. The lysosome as a command-and-control center for cellular metabolism. J Cell Biol. 2016;214(6):653–64. 27621362 10.1083/jcb.201607005 PMC5021098

[8] Zhu SY, Yao RQ, Li YX, et al. Lysosomal quality control of cell fate: a novel therapeutic target for human diseases. Cell Death Dis. 2020;11(9):817. 32999282 10.1038/s41419-020-03032-5 PMC7528093

[9] Kallunki T, Olsen OD, Jäättelä M. Cancer-associated lysosomal changes: friends or foes? Oncogene. 2013;32(16):1995–2004. 22777359 10.1038/onc.2012.292

[10] Pu J, Guardia CM, Keren-Kaplan T, Bonifacino JS. Mechanisms and functions of lysosome positioning. J Cell Sci. 2016;129(23):4329–39. 27799357 10.1242/jcs.196287 PMC5201012

[11] Fennelly C, Amaravadi RK. Lysosomal biology in cancer. Methods Mol Biol. 2017;1594:293–308. 28456991 10.1007/978-1-4939-6934-0_19 PMC5542621

[12] Piao S, Amaravadi RK. Targeting the lysosome in cancer. Ann N Y Acad Sci. 2016;1371(1):45–54. 26599426 10.1111/nyas.12953 PMC4879098

[13] Xu Y, Cao X, Zhang S, Zhang Y, Shen Z. High expression of LAMP1 as a prognostic marker in patients with epithelial ovarian cancer. Int J Clin Exp Pathol. 2017;10(8):9104–11. 31966783 PMC6965383

[14] Wang G, Ouyang B, Jing F, Dai X. GBA inhibition suppresses ovarian cancer growth, survival and receptor tyrosine kinase AXL-mediated signaling pathways. Korean J Physiol Pharmacol. 2023;27(1):21–9. 36575930 10.4196/kjpp.2023.27.1.21 PMC9806639

[15] Jin MZ, Jin WL. The updated landscape of tumor microenvironment and drug repurposing. Signal Transduct Target Ther. 2020;5:166. 32843638 10.1038/s41392-020-00280-x PMC7447642

[16] Dettmer J, Hong-Hermesdorf A, Stierhof YD, Schumacher K. Vacuolar H^+^-ATPase activity is required for endocytic and secretory trafficking in arabidopsis. Plant Cell. 2006;18(3):715–30. 16461582 10.1105/tpc.105.037978 PMC1383645

[17] Zhang Z, Yue P, Lu T, Wang Y, Wei Y, Wei X. Role of lysosomes in physiological activities, diseases, and therapy. J Hematol Oncol. 2021;14(1):79. 33990205 10.1186/s13045-021-01087-1 PMC8120021

[18] Zhou Y, Zhou B, Pache L, et al. Metascape provides a biologist-oriented resource for the analysis of systems-level datasets. Nat Commun. 2019;10. 10.1038/s41467-019-09234-6 PMC6447622 30944313

[19] Szklarczyk D, Gable AL, Nastou KC, et al. The STRING database in 2021: customizable protein–protein networks, and functional characterization of user-uploaded gene/measurement sets. Nucleic Acids Res. 2021;49(D1):D605. 33237311 10.1093/nar/gkaa1074 PMC7779004

[20] Jang JH. Principal component analysis of hybrid functional and vector data. Stat Med. 2021;40(24):5152–73. 34160848 10.1002/sim.9117 PMC9084921

[21] Iasonos A, Schrag D, Raj GV, Panageas KS. How to build and interpret a nomogram for cancer prognosis. JCO. 2008;26(8):1364–70. 10.1200/JCO.2007.12.9791 18323559

[22] Yoshihara K, Shahmoradgoli M, Martínez E, et al. Inferring tumour purity and stromal and immune cell admixture from expression data. Nat Commun. 4. 10.1038/ncomms3612 PMC3826632 24113773

[23] Chen B, Khodadoust MS, Liu CL, Newman AM, Alizadeh AA. Profiling tumor infiltrating immune cells with CIBERSORT. In: Methods in molecular biology (Clifton, NJ). 2018;1711:243. 10.1007/978-1-4939-7493-1_12 PMC5895181 29344893

[24] Mayakonda A, Lin DC, Assenov Y, Plass C, Koeffler HP. Maftools: efficient and comprehensive analysis of somatic variants in cancer. Genome Res. 2018;28(11):1747–56. 30341162 10.1101/gr.239244.118 PMC6211645

[25] Subramanian A, Tamayo P, Mootha VK, et al. Gene set enrichment analysis: a knowledge-based approach for interpreting genome-wide expression profiles. Proc Natl Acad Sci USA. 2005;102(43):15545–50. 16199517 10.1073/pnas.0506580102 PMC1239896

[26] Aykul S, Martinez-Hackert E. Determination of half-maximal inhibitory concentration using biosensor-based protein interaction analysis. Anal Biochem. 2016;508:97–103. 27365221 10.1016/j.ab.2016.06.025 PMC4955526

[27] Geeleher P, Cox N, Huang RS. pRRophetic: an R package for prediction of clinical chemotherapeutic response from tumor gene expression levels. PLoS ONE. 2014;9(9): e107468. 25229481 10.1371/journal.pone.0107468 PMC4167990

[28] Lheureux S, Gourley C, Vergote I, Oza AM. Epithelial ovarian cancer. Lancet. 2019;393(10177):1240–53. 30910306 10.1016/S0140-6736(18)32552-2

[29] Rousalova I, Krepela E. Granzyme B-induced apoptosis in cancer cells and its regulation (review). Int J Oncol. 2010;37(6):1361–78. 21042704 10.3892/ijo_00000788

[30] Wang J, Su X, Wang C, Xu M. Integrated analysis of prognostic immune-related genes in the tumor microenvironment of ovarian cancer. Ann Transl Med. 2022;10(2):91. 35282097 10.21037/atm-21-7014 PMC8848435

[31] Gao L, Ying F, Cai J, et al. Identification and validation of pyroptosis-related gene landscape in prognosis and immunotherapy of ovarian cancer. J Ovarian Res. 2023;16:27. 36707884 10.1186/s13048-022-01065-2 PMC9883900

[32] Wang D, Cao X, Zhang Y, et al. LAMP3 expression correlated with poor clinical outcome in human ovarian cancer. Tumour Biol. 2017;39(3):1010428317695014. 28349821 10.1177/1010428317695014

[33] Jiao J, Jiang L, Luo Y. N6-methyladenosine-related RNA signature predicting the prognosis of ovarian cancer. Recent Pat Anticancer Drug Discov. 2021;16(3):407–16. 34137363 10.2174/1574892816666210615164645

[34] Pils D, Tong D, Hager G, et al. A combined blood based gene expression and plasma protein abundance signature for diagnosis of epithelial ovarian cancer—a study of the OVCAD consortium. BMC Cancer. 2013;13:178. 23551967 10.1186/1471-2407-13-178 PMC3639192

[35] Hou P, Wan Q, Wang Q, Wu X, Lu X. Overexpression of RAB34 associates with tumor aggressiveness and immune infiltration in glioma. Biosci Rep. 2022;42(10):BSR2021624. 10.1042/BSR20212624 PMC9620491 36222286

[36] Goldenberg NM, Grinstein S, Silverman M. Golgi-bound Rab34 is a novel member of the secretory pathway. Mol Biol Cell. 2007;18(12):4762–71. 17881736 10.1091/mbc.E06-11-0991 PMC2096593

[37] Li X, Lu Y, Chen Y, Lu W, Xie X. MicroRNA profile of paclitaxel-resistant serous ovarian carcinoma based on formalin-fixed paraffin-embedded samples. BMC Cancer. 2013;13:216. 23627607 10.1186/1471-2407-13-216 PMC3648441

[38] Schlottmann F, Vera-Aviles M, Latunde-Dada GO. Duodenal cytochrome *b* (Cybrd1) ferric reductase functional studies in cells. Metallomics. 2017;9(10):1389–93. 28937159 10.1039/c7mt00254h

[39] Willis S, Villalobos VM, Gevaert O, et al. Single gene prognostic biomarkers in ovarian cancer: a meta-analysis. PLoS ONE. 2016;11(2): e0149183. 26886260 10.1371/journal.pone.0149183 PMC4757072

[40] Chen R, Cao J, Jiang W, Wang S, Cheng J. Upregulated expression of CYBRD1 predicts poor prognosis of patients with ovarian cancer. J Oncol. 2021;2021:7548406. 34594380 10.1155/2021/7548406 PMC8478559

[41] Nikolova DN, Doganov N, Dimitrov R, et al. Genome-wide gene expression profiles of ovarian carcinoma: Identification of molecular targets for the treatment of ovarian carcinoma. Mol Med Rep. 2009;2(3):365–84. 21475838 10.3892/mmr_00000109

[42] Gusev A, Lawrenson K, Lin X, et al. A transcriptome-wide association study of high-grade serous epithelial ovarian cancer identifies new susceptibility genes and splice variants. Nat Genet. 2019;51(5):815–23. 31043753 10.1038/s41588-019-0395-x PMC6548545

[43] Pharoah PDP, Tsai YY, Ramus SJ, et al. GWAS meta-analysis and replication identifies three new susceptibility loci for ovarian cancer. Nat Genet. 2013;45(4):362–70. 23535730 10.1038/ng.2564 PMC3693183

[44] Kreuzinger C, Geroldinger A, Smeets D, et al. A complex network of tumor microenvironment in human high-grade serous ovarian cancer. Clin Cancer Res. 2017;23(24):7621–32. 28972047 10.1158/1078-0432.CCR-17-1159

[45] Sabado RL, Balan S, Bhardwaj N. Dendritic cell-based immunotherapy. Cell Res. 2017;27(1):74–95. 28025976 10.1038/cr.2016.157 PMC5223236

[46] Ma Y, Shurin GV, Peiyuan Z, Shurin MR. Dendritic cells in the cancer microenvironment. J Cancer. 2012;4(1):36–44. 23386903 10.7150/jca.5046 PMC3564245

[47] Mu L, Sun B, Kong Q, et al. Disequilibrium of T helper type 1, 2 and 17 cells and regulatory T cells during the development of experimental autoimmune myasthenia gravis. Immunology. 2009;128(1 Pt 2):e826–36. 19740344 10.1111/j.1365-2567.2009.03089.x PMC2753914

[48] Kusuda T, Shigemasa K, Arihiro K, Fujii T, Nagai N, Ohama K. Relative expression levels of Th1 and Th2 cytokine mRNA are independent prognostic factors in patients with ovarian cancer. Oncol Rep. 2005;13(6):1153–8. 15870936

[49] Jiang Y, Wang C, Zhou S. Targeting tumor microenvironment in ovarian cancer: premise and promise. Biochim Biophys Acta Rev Cancer. 2020;1873(2): 188361. 32234508 10.1016/j.bbcan.2020.188361

[50] Hwang WT, Adams SF, Tahirovic E, Hagemann IS, Coukos G. Prognostic significance of tumor-infiltrating T-cells in ovarian cancer: a meta-analysis. Gynecol Oncol. 2012;124(2):192–8. 22040834 10.1016/j.ygyno.2011.09.039 PMC3298445

[51] Sato E, Olson SH, Ahn J, et al. Intraepithelial CD8^+^ tumor-infiltrating lymphocytes and a high CD8^+^/regulatory T cell ratio are associated with favorable prognosis in ovarian cancer. Proc Natl Acad Sci USA. 2005;102(51):18538–43. 16344461 10.1073/pnas.0509182102 PMC1311741

[52] Lundgren S, Berntsson J, Nodin B, Micke P, Jirström K. Prognostic impact of tumour-associated B cells and plasma cells in epithelial ovarian cancer. J Ovarian Res. 2016;9:21. 27048364 10.1186/s13048-016-0232-0 PMC4822228

[53] Kroeger DR, Milne K, Nelson BH. Tumor-infiltrating plasma cells are associated with tertiary lymphoid structures, cytolytic T-cell responses, and superior prognosis in ovarian cancer. Clin Cancer Res. 2016;22(12):3005–15. 26763251 10.1158/1078-0432.CCR-15-2762

[54] Zhang Q, Li H, Mao Y, et al. Apoptotic SKOV3 cells stimulate M0 macrophages to differentiate into M2 macrophages and promote the proliferation and migration of ovarian cancer cells by activating the ERK signaling pathway. Int J Mol Med. 2020;45(1):10–22. 31746376 10.3892/ijmm.2019.4408 PMC6889918

[55] Wang Y, Lyu Z, Qin Y, et al. FOXO1 promotes tumor progression by increased M2 macrophage infiltration in esophageal squamous cell carcinoma. Theranostics. 2020;10(25):11535–48. 33052231 10.7150/thno.45261 PMC7546008

[56] Zhang C, Li Z, Wang J, et al. Ethanol extracts of Solanum lyratum Thunb regulate ovarian cancer cell proliferation, apoptosis, and epithelial-to-mesenchymal transition (EMT) via the ROS-mediated p53 pathway. J Immunol Res. 2021;2021:5569354. 33869638 10.1155/2021/5569354 PMC8035038

[57] Yousefzadeh Y, Hallaj S, Baghi Moornani M, et al. Tumor associated macrophages in the molecular pathogenesis of ovarian cancer. Int Immunopharmacol. 2020;84: 106471. 32305830 10.1016/j.intimp.2020.106471

[58] Vitale I, Manic G, Coussens LM, Kroemer G, Galluzzi L. Macrophages and Metabolism in the Tumor Microenvironment. Cell Metab. 2019;30(1):36–50. 31269428 10.1016/j.cmet.2019.06.001

[59] Nowak M, Klink M. The role of tumor-associated macrophages in the progression and chemoresistance of ovarian cancer. Cells. 2020;9(5):1299. 32456078 10.3390/cells9051299 PMC7290435

[60] Galuppini F, Dal Pozzo CA, Deckert J, Loupakis F, Fassan M, Baffa R. Tumor mutation burden: from comprehensive mutational screening to the clinic. Cancer Cell Int. 2019;19:209. 31406485 10.1186/s12935-019-0929-4 PMC6686509

[61] Kim JY, Kronbichler A, Eisenhut M, et al. Tumor mutational burden and efficacy of immune checkpoint inhibitors: a systematic review and meta-analysis. Cancers (Basel). 2019;11(11):1798. 31731749 10.3390/cancers11111798 PMC6895916

[62] Goodman AM, Kato S, Bazhenova L, et al. Tumor mutational burden as an independent predictor of response to immunotherapy in diverse cancers. Mol Cancer Ther. 2017;16(11):2598–608. 28835386 10.1158/1535-7163.MCT-17-0386 PMC5670009

[63] Chalmers ZR, Connelly CF, Fabrizio D, et al. Analysis of 100,000 human cancer genomes reveals the landscape of tumor mutational burden. Genome Med. 2017;9:34. 28420421 10.1186/s13073-017-0424-2 PMC5395719

[64] Cui M, Xia Q, Zhang X, et al. Development and validation of a tumor mutation burden-related immune prognostic signature for ovarian cancers. Front Genet. 2022;12: 688207. 35087563 10.3389/fgene.2021.688207 PMC8787320

[65] Bi F, Chen Y, Yang Q. Significance of tumor mutation burden combined with immune infiltrates in the progression and prognosis of ovarian cancer. Cancer Cell Int. 2020;20:373. 32774167 10.1186/s12935-020-01472-9 PMC7405355

[66] Hafner A, Bulyk ML, Jambhekar A, Lahav G. The multiple mechanisms that regulate p53 activity and cell fate. Nat Rev Mol Cell Biol. 2019;20(4):199–210. 30824861 10.1038/s41580-019-0110-x

[67] Kruiswijk F, Labuschagne CF, Vousden KH. p53 in survival, death and metabolic health: a lifeguard with a licence to kill. Nat Rev Mol Cell Biol. 2015;16(7):393–405. 26122615 10.1038/nrm4007

[68] Lawrence MS, Stojanov P, Mermel CH, et al. Discovery and saturation analysis of cancer genes across 21 tumor types. Nature. 2014;505(7484):495–501. 24390350 10.1038/nature12912 PMC4048962

[69] Kandoth C, McLellan MD, Vandin F, et al. Mutational landscape and significance across 12 major cancer types. Nature. 2013;502(7471):333–9. 24132290 10.1038/nature12634 PMC3927368

[70] Rendell A, Thomas-Bland I, McCuish L, Taylor C, Binju M, Yu Y. Targeting tyrosine kinases in ovarian cancer: small molecule inhibitor and monoclonal antibody, where are we now? Biomedicines. 2022;10(9):2113. 36140214 10.3390/biomedicines10092113 PMC9495728

