# Supplementary material for: Development of a prognostic model based on lysosome-related genes for ovarian cancer: insights into tumor microenvironment, mutation patterns, and personalized treatment strategies
Source: Cancer Cell Int. 2024 Dec 19;24:419. doi: 10.1186/s12935-024-03586-w (PMC11661007; doi:10.1186/s12935-024-03586-w)
Supplement: Supplementary file 1 — Supplementary Material 1. [file 12935_2024_3586_MOESM1_ESM.docx]

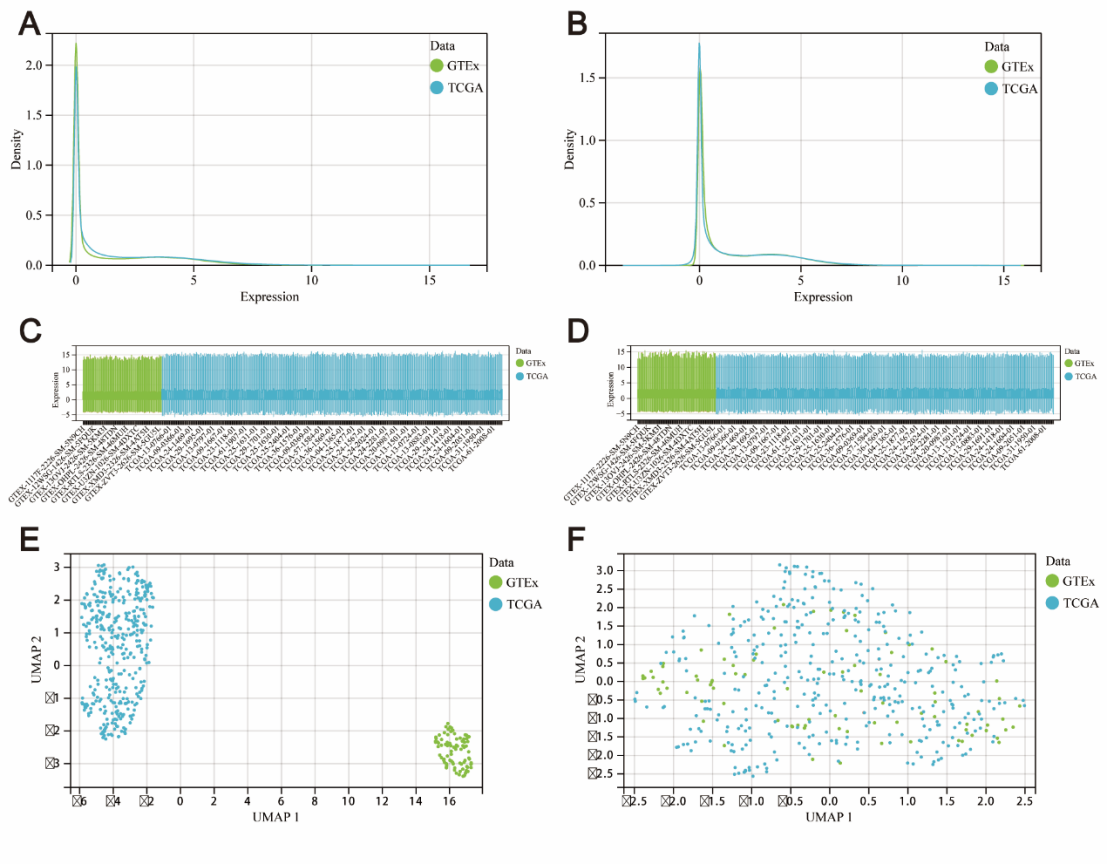


Supplementary Figure 1 presents the data distribution of both pre and post-removal of batch influences, visualized in the ensuing manner: (A, B) Density plots, (C, D) Boxplots, and (E, F) UMAP plots.


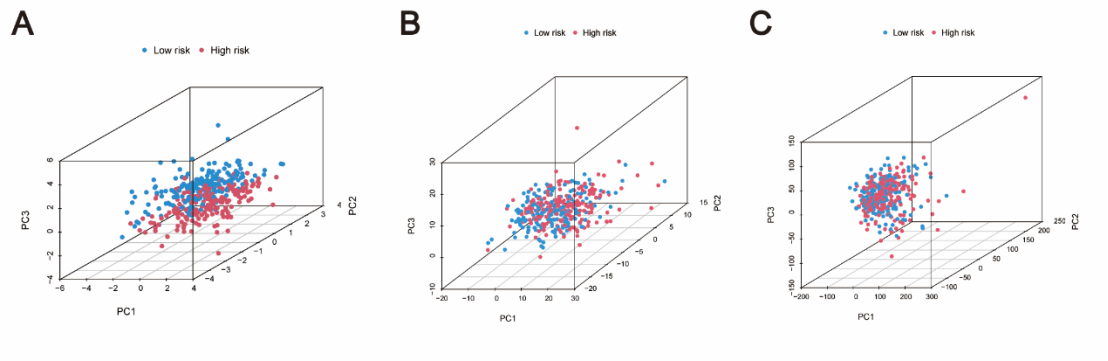


Supplementary Figure 2 displays the PCA executed for the low-risk versus high-risk cohorts, distinguished subsequently: (A) The prognostic model comprising a set of ten lysosomal genes. (B) The set of LRGs. (C) The complete set of all genes.
